# NF-κB-induced KIAA1199 promotes survival through EGFR signalling

**DOI:** 10.1038/ncomms6232

**Published:** 2014-11-04

**Authors:** Kateryna Shostak, Xin Zhang, Pascale Hubert, Serkan Ismail Göktuna, Zheshen Jiang, Iva Klevernic, Julien Hildebrand, Patrick Roncarati, Benoit Hennuy, Aurélie Ladang, Joan Somja, André Gothot, Pierre Close, Philippe Delvenne, Alain Chariot

**Affiliations:** 1 Interdisciplinary Cluster for Applied Genoproteomics (GIGA-Research) , University of Liege, 1, Avenue de l'hôpital, CHU, Sart-Tilman, Liege 4000, Belgium; 2 Laboratory of Medical Chemistry, University of Liege, 1, Avenue de l'hôpital, CHU, Sart-Tilman, Liege 4000, Belgium; 3 GIGA-Signal Transduction, University of Liege, 1, Avenue de l'hôpital, CHU, Sart-Tilman, Liege 4000, Belgium; 4 Laboratory of Experimental Pathology, University of Liege, 1, Avenue de l'hôpital, CHU, Sart-Tilman, Liege 4000, Belgium; 5 GIGA-Cancer, University of Liege, 1, Avenue de l'hôpital, CHU, Sart-Tilman, Liege 4000, Belgium; 6 GIGA Transcriptomics Facility, University of Liege, 1, Avenue de l'hôpital, CHU, Sart-Tilman, Liege 4000, Belgium; 7 GIGA-Infection, Immunity and Inflammation, Department of Medicine/Hematology, University of Liege, CHU, Sart-Tilman, Liege 4000, Belgium; 8 Walloon Excellence in Life Sciences and Biotechnology (WELBIO) , University of Liege, 1, Avenue de l'hôpital, CHU, Sart-Tilman, Liege 4000, Belgium

## Abstract

Constitutive activation of EGFR- and NF-κB-dependent pathways is a hallmark of cancer, yet signalling proteins that connect both oncogenic cascades are poorly characterized. Here we define *KIAA1199* as a BCL-3- and p65-dependent gene in transformed keratinocytes. KIAA1199 expression is enhanced on human papillomavirus (HPV) infection and is aberrantly expressed in clinical cases of cervical (pre)neoplastic lesions. Mechanistically, KIAA1199 binds Plexin A2 and protects from Semaphorin 3A-mediated cell death by promoting EGFR stability and signalling. Moreover, KIAA1199 is an EGFR-binding protein and KIAA1199 deficiency impairs EGF-dependent Src, MEK1 and ERK1/2 phosphorylations. Therefore, EGFR stability and signalling to downstream kinases requires KIAA1199. As such, KIAA1199 promotes EGF-mediated epithelial–mesenchymal transition (EMT). Taken together, our data define KIAA1199 as an oncogenic protein induced by HPV infection and constitutive NF-κB activity that transmits pro-survival and invasive signals through EGFR signalling.

## Abstract (web summary)

The cross-talk between constitutively active EGFR- and NF-κB-dependent pathways in cancer is poorly understood. Here, the authors identify KIAA1199 as a BCL3 and NF-κB-regulated protein that is expressed in cervical lesions and promotes tumorigenesis through Plexin A2 binding and regulation of EGFR stability.

## Graphical abstract

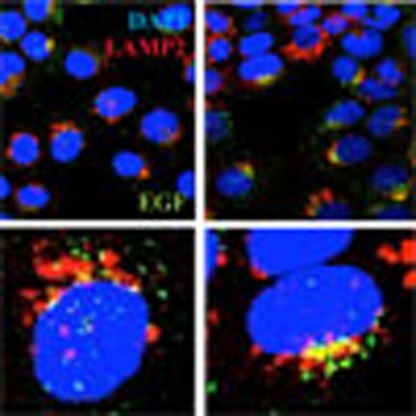

A better understanding of the mechanisms underlying tumour initiation and progression requires an exhaustive characterization of signalling pathways involved in cell proliferation and survival. Among them are nuclear factor-κB (NF-κB)-activating cascades, which are critical in inflammation and immunity when properly regulated^1^. Aberrant activation of NF-κB transcription factors is correlated to cancer, as they drive the expression of anti-apoptotic genes, cyclins and proto-oncogenes, and also promotes angiogenesis and metastasis^2^. Yet, the contribution of particular NF-κB subunits in tumour development remains poorly understood.

Studies that addressed the roles of NF-κB in normal epidermis and in skin cancers led to conflicting results. Indeed, although enhanced NF-κB activities were reported in squamous cell carcinomas, inhibition of this pathway in normal epidermis paradoxically promoted cell carcinoma development^3^,^4^,^5^,^6^. Similarly, despite a constitutive NF-κB activity seen in cancer of the uterine cervix^7^,^8^,^9^,^10^, the precise role of NF-κB in the development of these tumours, which are associated with human papillomavirus (HPV) infection, also remains unclear^11^,^12^. Indeed, although HPV16 E6 and E7 proteins promote cell proliferation and survival by inactivating p53 and Rb tumour suppressor functions, conflicting data as to whether these viral products activate or repress NF-κB in cervical epithelial cells have been reported^13^,^14^,^15^,^16^,^17^,^18^. Therefore, further studies are needed to clarify the mechanistic link between NF-κB and keratinocyte transformation in the skin and cervix.

The oncogenic potential of NF-κB relies on p50 and p65, as well as on BCL-3, a nuclear IκB protein originally identified through molecular cloning of the breakpoint of the t(14;19) chromosomal translocation found in a subset of human B-cell chronic lymphocytic leukemias^19^. BCL-3 is overexpressed in a variety of haematological tumours and is oncogenic, as evidenced by its ability to transform NIH3T3 cells, to induce MDM2 and to protect from ultraviolet-mediated apoptosis^19^,^20^,^21^,^22^,^23^,^24^,^25^. Aberrant BCL-3 expression has also been reported in breast, nasopharyngeal and prostate cancers, and in hepatocarcinomas^26^,^27^,^28^,^29^. Finally, increased nuclear BCL-3 levels cause enhanced keratinocyte proliferation in familial cylindromatosis, a genetic disease characterized by benign tumours of hair-follicle keratinocytes that results from loss-of-function mutations of CYLD, a deubiquitinating enzyme limiting BCL-3 nuclear levels^30^,^31^.

We show here that BCL-3 induces expression of *KIAA1199* in immortalized and transformed keratinocytes. KIAA1199 is a poorly characterized protein whose expression is enhanced in breast, gastric and colon cancer^32^,^33^,^34^,^35^,^36^. KIAA1199 promotes hyaluronan depolymerization in skin fibroblasts^37^. Cell migration relies on KIAA1199, but signalling pathways in which KIAA119 is acting only start to be elucidated^32^. KIAA1199 appears to mediate endoplasmic reticulum calcium leakage, which results in cell motility through protein kinase Cα activation^32^. These data suggest that KIAA1199 is an oncogenic protein but it is currently unclear whether KIAA1199 promotes or limits cell death^38^. We demonstrate here that levels of KIAA1199 are increased in HPV-positive cells and upregulated in cervical (pre)neoplastic lesions. KIAA1199 associates with Plexin A2 and protects from Semaphorin 3A-dependent cell death, at least by promoting epidermal growth factor receptor (EGFR) stability and signalling. Tumour necrosis factor-α (TNFα)-mediated cell apoptosis is also negatively regulated by KIAA1199. Moreover, KIAA1199, as an EGFR-binding protein, promotes EGF-mediated EGFR, Src and MEK1 phosphorylations, thus suggesting that KIAA1199 connects EGFR to downstream kinases. As a result, EGF-induced epithelial–mesenchymal transition (EMT) also requires KIAA1199. Therefore, KIAA1199 links HPV and constitutive NF-κB activation to cell survival and invasion by counteracting Semaphorin 3A-mediated cell death and by promoting EGFR signalling.

## Results

### KIAA1199 is a BCL-3- and p65-induced protein

**Figure 1:**
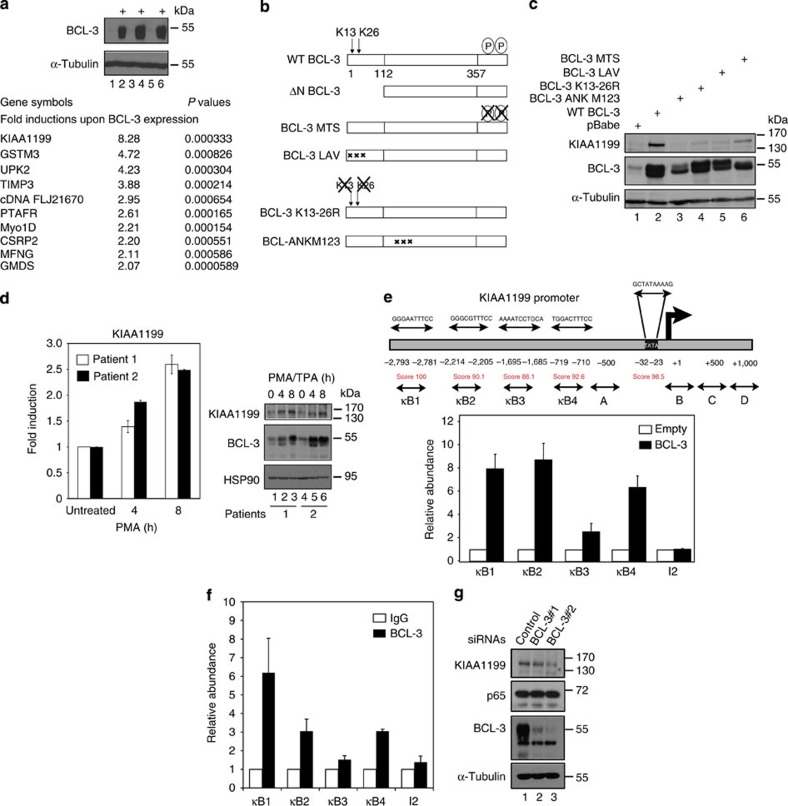
KIAA1199 is a BCL-3-induced gene in immortalized keratinocytes. (**a**) Identification of BCL-3-induced genes. Total RNAs from three distinct infections of control or BCL-3-overexpressing HaCat cells were subjected to microarrays. The ten most induced candidates are listed (*P*-values by Student’s *t*-test). (**b**) Representation of wild type (WT) BCL-3 and mutants^20^,^40^,^61^. (**c**) BCL-3 integrity is required for KIAA1199 expression. HaCat cells were infected with a control retrovirus (pBabe) or with the indicated retroviral constructs, and cell extracts were subjected to western blotting (WB) analyses. (**d**) BCL-3 and KIAA1199 expressions are induced by PMA/TPA (200 ng ml^−1^) in primary foreskin keratinocytes. On the left, total RNAs from untreated or PMA/TPA-stimulated primary foreskin keratinocytes of two healthy donors (patients 1 and 2) were subjected to real-time PCR to assess KIAA1199 mRNA levels. mRNA levels in untreated cells was set to 1 and levels in other experimental conditions were relative to that after normalization with 18S rRNA. Data from two independent experiments (means±s.d.) are shown. On the right, WBs were carried out on cell extracts from untreated or PMA/TPA-stimulated primary foreskin keratinocytes. (**e**) Recruitment of BCL-3 to the *KIAA1199* promoter. Sequences of κB sites identified on the *KIAA1199* promoter are represented with their scores (TFSEARCH software). ChIP assays were conducted with an anti-BCL-3 antibody and extracts from control (‘Empty’) or BCL-3-overexpressing HaCat cells. Associated DNA was analysed by real-time PCR using primers spanning the κB sites. BCL-3 density on every κB sites and on intron 2 (‘I2’) in control cells was set to 1 and other values are expressed relative to that. Primers within intron 2 were used as negative control. Data obtained from seven ChIP assays performed on five distinct cultures of control or BCL-3-overexpressing HaCat cells are shown. Error bars denote s.d. (**f**) Recruitment of endogenous BCL-3 to the *KIAA1199* promoter. ChIP assays were conducted with an anti-BCL-3 or -IgG (negative control) antibody and extracts from CaSki cells. Associated DNA was analysed as described in **e**. BCL-3 density on every κB sites as well as on intron 2 (‘I2’) was relative to signals obtained with the anti-IgG antibody. Data from two ChIP assays done in triplicates are shown. Error bars denote s.d. (**g**) BCL-3 controls KIAA1199 expression in cervical cancer cells. CaSki cells were transfected with the indicated siRNAs and extracts were subjected to WBs.

NF-κB contributes to the survival and growth of immortalized but not tumorigenic human keratinocytes HaCat cells. As this cell line is a model in which deregulated NF-κB signalling supports malignant traits^39^, we selected it to gain insights into mechanisms underlying BCL-3 oncogenic potential. Transcriptomic analyses were conducted with total RNAs from control or BCL-3-overexpressing HaCat cells. The most intensively induced candidate was *KIAA1199* (Fig. 1a). BCL-3 regulates gene transcription through two domains, including a region located upstream the ankyrin repeats (Fig. 1b). This domain was critical for the induction of KIAA1199 expression as the ΔN BCL-3 mutant failed to do so in HaCat cells (Supplementary Fig. 1A). Similar results were obtained on expression of the BCL3 ANK M1,2,3, unable to bind p50 and p52 NF-κB proteins^40^ (Fig. 1c). Moreover, both lysines 13 and 26, the CtBP-binding motif, as well as the carboxy-terminal GSK3-phosphorylated sites of BCL-3 are also required, as KIAA1199 expression was barely detectable in HaCat cells that overexpress BCL-3 K13-26R, BCL-3 LAV or the BCL-3 MTS mutant, respectively (Fig. 1c). Therefore, BCL-3 integrity is required to promote KIAA1199 expression. As PMA/TPA increases nuclear BCL-3 levels in keratinocytes^30^, we next wondered whether PMA/TPA positively regulates KIAA1199 levels in HaCat cells. Both BCL-3 and KIAA1199 expressions were indeed similarly induced on PMA/TPA treatment in HaCat cells as well as in primary foreskin keratinocytes from two healthy donors (Supplementary Fig. 1B and Fig. 1d, respectively).

The *KIAA1199* promoter has four κB sites upstream the TATA box. BCL-3 was recruited to κB sites 1, 2 and 4, and to a less extent to κB site 3, as evidenced by chromatin immunoprecipitation (ChIP) assays (Fig. 1e). Interestingly, endogenous BCL-3 was also recruited on the same κB sites of *KIAA1199* promoter in cervical cancer-derived CaSki cells (Fig. 1f). Consistently, KIAA1199 expression relies on BCL-3 in cervical cancer-derived cells as small interfering RNA (siRNA)-mediated BCL-3 depletion impaired KIAA1199 expression in CaSki cells (Fig. 1g). Moreover, BCL-3 depletion in CaSki cells also impaired histone acetylation (H3K9) at the *KIAA1199* promoter, but not in the downstream region (Supplementary Fig. 1C). Although HPV-positive SiHa cells did not show high KIAA1199 protein levels, KIAA1199 messenger RNA levels nevertheless decreased on BCL-3 deficiency in these cells (Supplementary Fig. 2A), thus demonstrating that BCL-3 drives KIAA1199 expression in all tested HPV-positive cervical cancer-derived cell lines.

As BCL-3 regulates gene transcription when bound to p50 and p52, and because the NF-κB subunit p65 also binds p50 to drive expression of target genes, we next explored whether KIAA1199 expression also relies on p65. Endogenous p65 was indeed recruited to the same κB sites as BCL-3 in CaSki cells (Supplementary Fig. 2B). Moreover, KIAA1199 expression was decreased on p65 depletion in both CaSki and SiHa cells (Supplementary Fig. 2C,D, respectively). Of note, we systematically noticed a slight increase in BCL-3 levels in p65-depleted CaSki cells (Supplementary Fig. 2C). p65 depletion also impaired KIAA1199 expression in BCL-3-overexpressing HaCat cells (Supplementary Fig. 3A). Moreover, histone acetylation (H3K9) at the *KIAA1199* promoter was decreased on p65 deficiency in CaSki cells, as evidenced by ChIP assays (Supplementary Fig. 3B). Finally, the recruitment of RNA polymerase II on the *KIAA1199* promoter was also defective on BCL-3 or p65 deficiency in CaSki cells (Supplementary Fig. 4). Yet, TNFα-induced interleukin-8 but not KIAA1199 expression in HaCat, SiHa, as well as in BCL-3-expressing CaSki cells, suggesting that *KIAA1199* is not a target gene of the classical NF-κB-activating pathway, which relies on the p50/p65 heterodimer (Supplementary Figs. 5A–C). Thus, both BCL-3 and p65 drive KIAA1199 expression in immortalized and transformed keratinocytes.

### Increased KIAA1199 expression in HPV-positive cells

**Figure 2:**
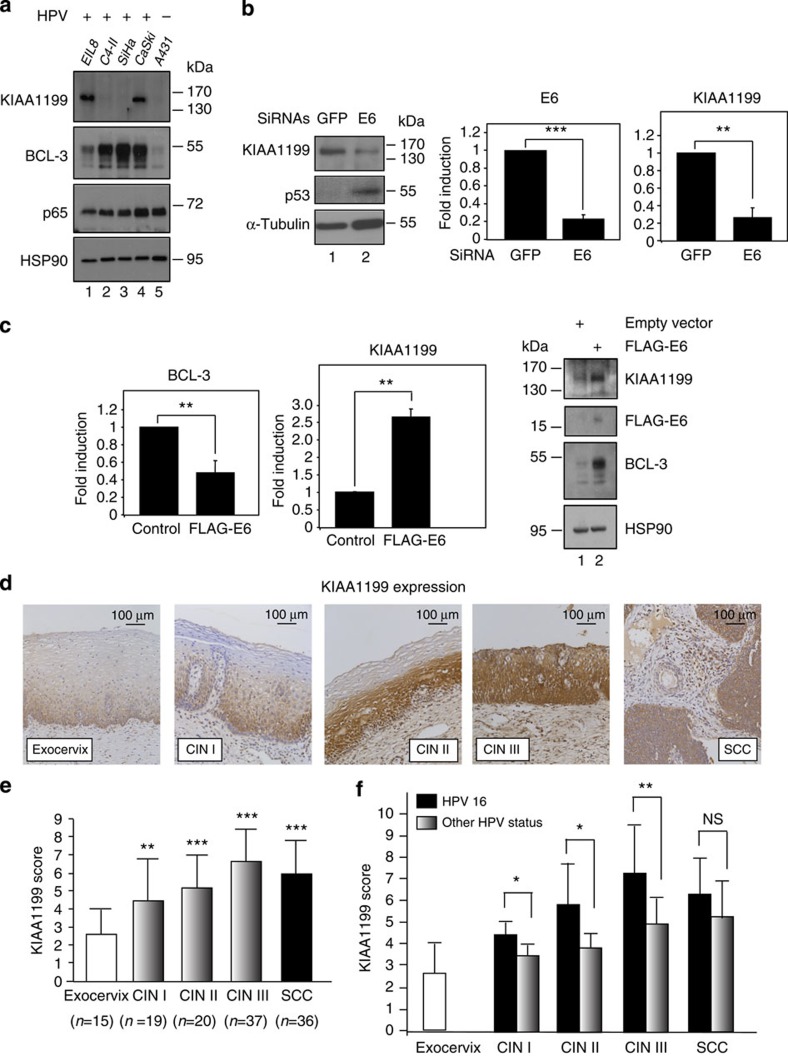
KIAA1199 expression correlates with HPV status in cervical cancer-derived cells and with cervical intraepithelial neoplasia progression. (**a**) KIAA1199, BCL-3 and p65 expression levels in HPV-positive and -negative cervical cancer-derived cell lines. Anti-KIAA1199, -BCL-3 and -p65 WBs using protein extracts from the indicated cell lines are shown. (**b**) E6 depletion in a HPV16-positive cervical carcinoma-derived cell line decreases KIAA1199 mRNA levels. CaSki cells were transfected with the indicated siRNAs (E6 siRNA pool) and the resulting cells were subjected to real-time PCR to assess E6 or KIAA1199 mRNA levels. The abundance of E6 and KIAA1199 transcripts in cells transfected with the control siRNA (‘GFP’) was set to 1 and their levels in cells transfected with the other siRNAs were relative to that after normalization with 18S rRNA. Data from three independent experiments (means±s.d.) are shown (Student’s *t*-test *P*-values: **<0.01). (**c**) Ectopic HPV16 E6 expression in a HPV16-negative cell line enhances KIAA1199 expression. A431 cells were transfected with a control or an E6-expressing construct and cell extracts from the resulting cells were subjected to real-time PCR or to WB analyses. The abundance of BCL-3 and KIAA1199 transcripts in cells transfected with the control expression plasmid (‘empty vector’) was set to 1 and their levels in E6-expressing cells were relative to that after normalization with β-actin. Data from two real-time PCR analyses performed in triplicates (means±s.d.) are shown (Student’s *t*-test *P*-values: **<0.01). (**d**) KIAA1199 immunostaining in normal exocervix, cervical intraepithelial neoplasia grade I (CIN I), grade II (CIN II), grade III (CIN III) and invasive squamous cell carcinoma (SCC) areas. Original magnifications: × 200. (**e**) Semi-quantitative evaluation of KIAA1199 expression in these biopsies (see Methods for details). Asterisks indicate statistically significant differences compared with normal exocervix (Student’s *t*-test *P*-values: ***<0.001; **<0.01). (**f**) KIAA1199 expression is significantly higher in (pre)neoplastic lesions transformed by HPV16 compared with lesions associated with other HPV types. Semi-quantitative evaluation of KIAA1199 expression in normal exocervix, cervical intraepithelial neoplasia grade I (CIN I), grade II (CIN II), grade III (CIN III) and invasive squamous cell carcinoma (SCC) areas. Asterisks indicate statistically significant differences between HPV16-positive cases and cases infected with other HPV types (Student’s *t*-test *P*-values: **<0.01; *<0.05).

While addressing KIAA1199 and BCL-3 expressions in cervical cancer cells, we noticed that BCL-3 protein levels were higher in HPV-positive EIL8 and CaSki cells than in the HPV-negative A431 cell line (Fig. 2a). As HPV16 E6 inactivates CYLD^41^ and because defective CYLD causes enhanced nuclear import of BCL-3 (ref. 30), we reasoned that HPV16 E6 may induce *KIAA1199* expression through BCL-3 in transformed keratinocytes. As HPV type 16 or 18 infection leads to E6 expression and to p53 degradation^42^, we depleted E6 in CaSki cells and addressed KIAA1199 expression. As expected, E6 depletion restored p53 expression (Fig. 2b). Importantly, a fivefold reduction of E6 expression caused a similar decrease in KIAA1199 mRNA levels (Fig. 2b), thus suggesting that HPV16 infection drives KIAA1199 expression. Ectopic E6 expression enhanced protein but not mRNA levels of BCL-3 in the HPV-negative A431 cells. As a result, KIAA1199 mRNA and protein levels were increased (Fig. 2c). Thus, HPV16 positively regulates BCL-3 and KIAA1199 levels in cervical cancer-derived cells. We next assessed KIAA1199 expression in exocervix and in distinct grades of cervical intraepithelial neoplasia (CIN) or squamous cell carcinoma by immunohistochemistry. The normal exocervical epithelium only showed marginal KIAA1199 staining, whereas an intermediate or high KIAA1199 expression was detected in (pre)neoplastic lesions (Fig. 2d). Indeed, KIAA1199 protein levels positively correlated with CIN progression but did not further increase in squamous cell carcinoma (Fig. 2e). Importantly, KIAA1199 levels were significantly higher in HPV16-positive (pre)neoplastic lesions compared with those associated with other HPV subtypes (Fig. 2f). Therefore, KIAA1199 expression is positively correlated with HPV status of cervical (pre)neoplastic lesions.

### KIAA1199 protects from Semaphorin 3A-mediated cell death

**Figure 3:**
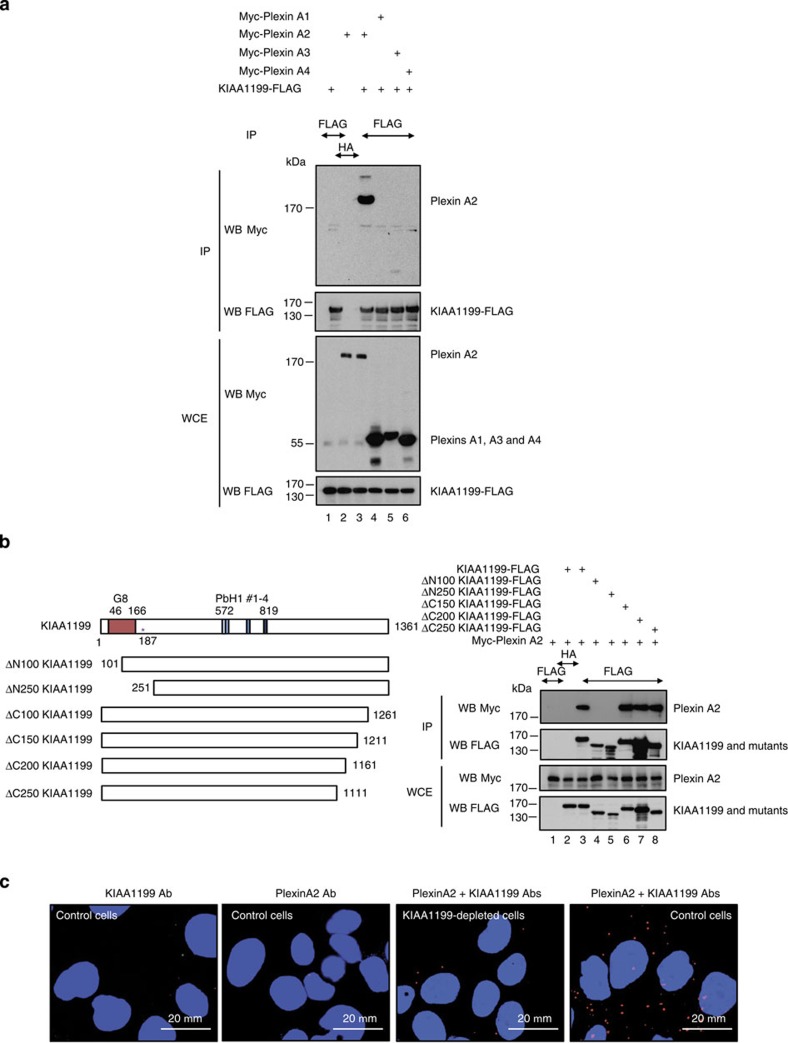
KIAA1199 is a Plexin A2-binding protein. (**a**) KIAA1199 binds Plexin A2 but not Plexin A1, A3 and A4. HEK293 cells were transfected with the indicated expression plasmids and anti-FLAG and -HA (negative control) immnunoprecipitations (IP) followed by anti-Myc and -FLAG WBs were carried out, as indicated. Whole cell extracts (WCE) were subjected to anti-Myc and -FLAG WBs as well (bottom panels). (**b**) The first 100 N-terminal amino acids of KIAA1199 are required to bind Plexin A2. HEK293 cells were transfected with the indicated expression plasmids and the resulting cells extracts were subjected to IP and WB analyses as described here before (panels on the right). KIAA1199 mutants tested in IPs are schematically represented on the left. PbH1, parallel β-helix helix domains. The asterisk denotes the Arg187 residue required for Hyaluronan-degrading activity^37^. (**c**) Plexin A2 binds KIAA1199 at the endogenous level in CaSki cells. Proximal ligation assays (PLA) were carried out in control CaSki cells to visualize the binding of Plexin A2 to KIAA1199 (red dots). The assay was also carried out in KIAA1199-depleted CaSki cells as an additional negative control.

As Plexin A2 has been identified as a KIAA1199-interacting protein through yeast two-hybrid experiments^43^, we conducted co-immunoprecipitations and showed that Myc-Plexin A2, but not the cytoplasmic tails of Plexin A1, A3 and A4, bound KIAA1199-FLAG in HEK293 cells (Fig. 3a). KIAA1199 harbours a G8 domain that includes eight conserved Gly residues in five β-strand pairs as well as four PbH1 motifs^34^,^44^. The integrity of the G8 domain of KIAA1199 was required for binding to Plexin A2, as the ΔN100 KIAA1199 mutant failed to associate to this receptor (Fig. 3b). On the other hand, KIAA1199 mutants lacking up to 250 amino acids at the C-terminal end still bound Plexin A2 (Fig. 3b). Therefore, KIAA1199 binds Plexin A2 through an amino-terminal region that includes the G8 domain. We next conducted proximal ligation assays and demonstrated that KIAA1199 bound Plexin A2 at the endogenous level (Fig. 3c).

**Figure 4:**
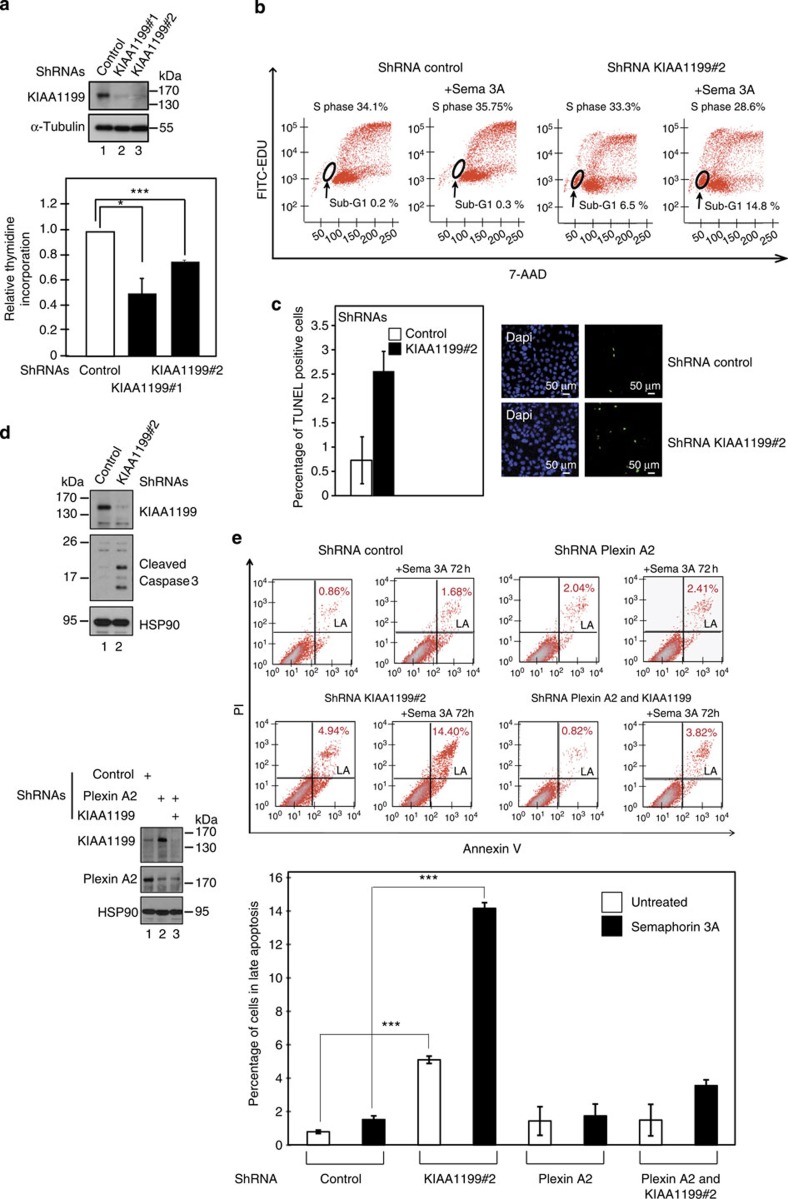
KIAA1199 protects from Semaphorin 3A-mediated cell death. (**a**) KIAA1199-depleted cells do not efficiently incorporate thymidine. On the top, WBs to assess KIAA1199 and α-tubulin in extracts from control (lane 1) or KIAA1199-depleted CaSki cells (lanes 2 and 3). At the bottom, thymidine incorporation assays were conducted on control versus KIAA1199-depleted CaSki cells. Incorporation in control cells was set to 1 and values obtained in other experimental conditions were relative to that. Data obtained from four independent experiments performed in triplicates are shown. Error bars denote s.d. (*P<0.05, ***P<0.001, Student’s *t*-test). (**b**) KIAA1199 protects cervical cancer cells from Semaphorin 3A-mediated cell death. Control or KIAA1199-depleted (shRNA KIAA1199#2) CaSki cells were untreated or stimulated with Semaphorin 3A (100 ng ml^−1^) for 24 h and FACS analysis were conducted to quantity cells in S or in G1 phases. 7-AAD, 7-aminoactinomycin D, used as a marker of DNA content. (**c**) Increased number of TUNEL-positive cells on KIAA1199 deficiency. Cells undergoing cell death (TUNEL-positive cells) were quantified in control or KIAA1199-depleted CaSki cells. The histogram shows the percentage of TUNEL-positive cells per field. Quantification, using the ImageJ software, was done with five blindy taken fields from two distinct experiments. DAPI staining was carried out to visualize the nuclei. Error bars denote s.d. (**d**) Caspase 3 activation on KIAA1199 deficiency. Control or KIAA1199-deficient CaSki cells were subjected to WB analyses, using the indicated antibodies. Cleaved forms of caspase 3 (19 and 15 kDa fragments) appear on KIAA1199 deficiency. (**e**) Plexin A2 depletion prevents cell apoptosis in KIAA1199-deficient CaSki cells. Control, Plexin A2 or Plexin A2 and KIAA1199-depleted CaSki cells were left untreated or stimulated with Semaphorin 3A (100 ng ml^−1^) for 72 h and FACS analysis were conducted to quantify cells in late apoptosis (LA). WBs were also conducted to assess Plexin A2 and KIAA1199 protein levels in all experimental conditions. At the bottom, FACS data from two independent experiments are also illustrated in the histogram (Student *t*-test, *P*-values: ***<0.001).

As Plexin A2 transduces signals on binding of class 3 Semaphorins to neuropilins^45^, we explored whether KIAA1199 is acting in the Semaphorin 3A- and Plexin A2-dependent pathway. All Plexin members were detected at the mRNA levels in both CaSki and HaCat cells (with the exception of Plexin C1 in HaCat cells) (Supplementary Fig. 6). KIAA1199-depleted CaSki cells did not efficiently incorporate thymidine (Fig. 4a), which reflects proliferation defects and/or enhanced cell death. Although the percentage of cells in S phase did not significantly change (from 34.1% to 33.3%), cells in sub-G1 phase increased from 0.2% up to 6.5% in KIAA1199-depleted CaSki cells (Fig. 4b), suggesting that KIAA1199 acts as a pro-survival protein. KIAA1199 deficiency indeed correlated with an increase of TUNEL (TdT-mediated dUTP nick end labelling)-positive cells and with the appearance of cleaved forms of Caspase 3 (Fig. 4c,d, respectively).

As prolonged stimulation with class 3 Semaphorins triggers apoptosis in cancer cells^46^, we assessed Semaphorin 3A-mediated cell death in control versus KIAA1199-depleted CaSki cells. KIAA1199-deficient cells (14.8%) were in sub-G1 phase on stimulation with Semaphorin 3A for 72 h (Fig. 4b). Consistently, 14.40% of KIAA1199-depleted cells were in late apoptotic phase in contrast to only 1.68% of control cells after 72 h of stimulation (Fig. 4e). Similar results were obtained by using another short hairpin RNA (shRNA) lentiviral construct to deplete KIAA1199 (Supplementary Fig. 7). Of note, the percentage of KIAA1199-depleted cells in apoptosis was increasing in a time-dependent manner (Supplementary Fig. 7). Therefore, KIAA1199 protects cervical cancer cells from Semaphorin 3A-mediated cell death. As Semaphorin 3A signals through Plexin A2, we next explored whether cell death relies on this receptor. Depletion of Plexin A2 protected CaSki cells from Semaphorin 3A-mediated cell death (Fig. 4e). Of note, KIAA1199 levels were increased on Plexin A2 deficiency (Fig. 4e). Importantly, Semaphorin 3A-mediated cell death as a result of KIAA1199 deficiency was abolished on co-depletion of Plexin A2, as only 3.82% of those cells were undergoing late apoptosis (Fig. 4e). Therefore, KIAA1199 protects cells from Semaphorin 3A- and Plexin A2-dependent cell death in cervical cancer-derived cells.

### KIAA1199 links Semaphorin 3A to EGFR phosphorylation

**Figure 5:**
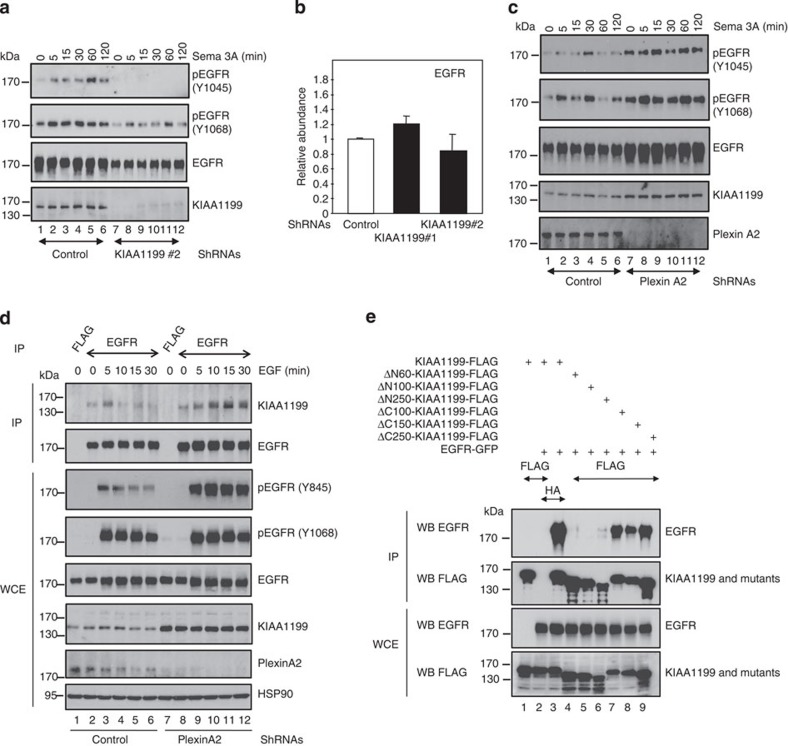
KIAA1199 connects Semaphorin 3A signalling to EGFR phosphorylation. (**a**) Semaphorin 3A-mediated EGFR phosphorylation requires KIAA1199. Control or KIAA1199-depleted CaSki cells were untreated or stimulated with Semaphorin 3A (100 ng ml^−1^) and WB analyses were carried out on the resulting cell extracts (lysis in SDS 1%). (**b**) KIAA1199 deficiency does not have an impact on EGFR mRNA levels in cervical cancer cells. Total RNAs from control or KIAA1199-depleted (shRNA KIAA1199#1 or shRNA KIAA1199#2) CaSki cells were subjected to real-time PCR, to assess EGFR mRNA levels. The abundance of transcripts in control cells was set to 1 and their levels in KIAA1199-depleted cells were relative to that after normalization with glyceraldehyde 3-phosphate dehydrogenase (GAPDH). Data from two independent experiments (means±s.d.) are shown. (**c**) Plexin A2 deficiency potentiates Semaphorin 3A-mediated EGFR phosphorylation. Control or Plexin A2-depleted CaSki cells were left untreated or stimulated with Semaphorin 3A for the indicated periods of time. The resulting cell extracts (lysis in SDS 1%) were subjected to WBs using the indicated antibodies. (**d**) Plexin A2 deficiency prolongs the binding of KIAA1199 to EGFR on EGF stimulation. Control or Plexin A2-deficient cells were untreated or stimulated with EGF for the indicated periods of time. Cell extracts were subjected to anti-FLAG (negative control) or -EGFR immunoprecipitations followed by anti- KIAA1199 or -EGFR WBs (top panels). Crude cell extracts were subjected to anti-pEGFR (Y845 and Y1068) (to validate the triggering of the EGF-dependent pathway), -EGFR, -KIAA1199, -Plexin A2 and -HSP90 WBs, as indicated. (**e**) KIAA1199 binds EGFR through its N-terminal domain. Cells were transfected with the indicated expression plasmids and protein extracts were subjected to anti-HA (negative control) or -FLAG IPs followed by an anti-EGFR WB (top panel). Crude cell extracts were also subjected to anti-EGFR and -FLAG WB analyses (bottom panels).

As some Plexins bind and activate receptor tyrosine kinases (RTKs) such as MET, VEGFR2 and HER-2 to transmit Semaphorin-dependent signals in cancer cells^47^,^48^, we next explored whether Semaphorin 3A triggers EGFR phosphorylation and, if so, whether KIAA1199 is required for this cross-talk. Semaphorin 3A indeed transiently triggered EGFR phosphorylation on Tyr residues 1045 (Y1045) and 1068 (Y1068) (Fig. 5a). Importantly, Semaphorin 3A-dependent EGFR phosphorylation on both tyrosine residues was severely impaired on KIAA1199 deficiency, at least due to decreased EGFR expression at the protein but not mRNA level (Fig. 5a,b). Therefore, KIAA1199 connects Semaphorin 3A signalling to EGFR phosphorylation and maintains high EGFR levels in cervical cancer-derived cells. Taken together, our data suggest that KIAA1199 promotes EGFR stability and signalling.

To better understand the contribution of Plexin A2 in this pathway, we assessed Semaphorin 3A-dependent EGFR phosphorylation in Plexin A2-depleted CaSki cells and noticed that EGFR phosphorylation on Tyr residues 1045 and 1068 was enhanced, at least due to increased EGFR levels (Fig. 5c). Consistently, KIAA1199 levels were also enhanced in Plexin A2-depleted CaSki cells (Fig. 5c), further supporting the notion that KIAA1199 and EGFR protein levels are positively correlated.

### KIAA1199 binds EGFR

Given the fact that KIAA1199 and EGFR protein levels are positively correlated and because KIAA1199 is required to promote EGFR phosphorylation through the Semaphorin 3A-dependent pathway, we next wondered whether KIAA1199 binds EGFR and, if so, whether such interaction is modulated by EGF, an EGFR-activating signal. KIAA1199 was indeed found in anti-EGFR immunoprecipitates in both untreated and EGF-stimulated CaSki cells and this interaction weakened after 30 min (Fig. 5d). Interestingly, such interaction was sustained on EGF stimulation upon Plexin A2 deficiency, most probably as a result of increased KIAA1199 levels (Fig. 5d). Although basal levels of phosphorylated EGFR (pEGFR) on Tyr residues 1068 were slightly increased on Plexin A2 deficiency, pEGFR levels on Tyr residues 1068 on EGF stimulation did not rely on Plexin A2 (Fig. 5d). On the other hand, EGF-dependent EGFR phosphorylation on Tyr 845 was enhanced on Plexin A2 deficiency (Fig. 5d). Therefore, our data suggest that Plexin A2 limits EGFR stability and signalling, at least by negatively regulating KIAA1199 levels.

To learn more on KIAA1199 domains required for binding to EGFR, we generated additional KIAA1199 mutants that lack N-terminal amino acids and tested their association with EGFR by co-immunoprecipitations. The deletion of the first N-terminal 60 amino acids of KIAA1199 impaired its binding to EGFR (Fig. 5e). Consistently, the ΔN100- and ΔN250-KIAA1199 mutants also failed to bind EGFR (Fig. 5e). On the other hand, the C-terminal part of KIAA1199 was dispensable for the binding to EGFR as ΔC100-, ΔC150- and ΔC250-KIAA1199 mutants still bound this receptor (Fig. 5e). Thus, the N-terminal part of KIAA1199 that includes the G8 domain is critical for binding to EGFR.

**Figure 6:**
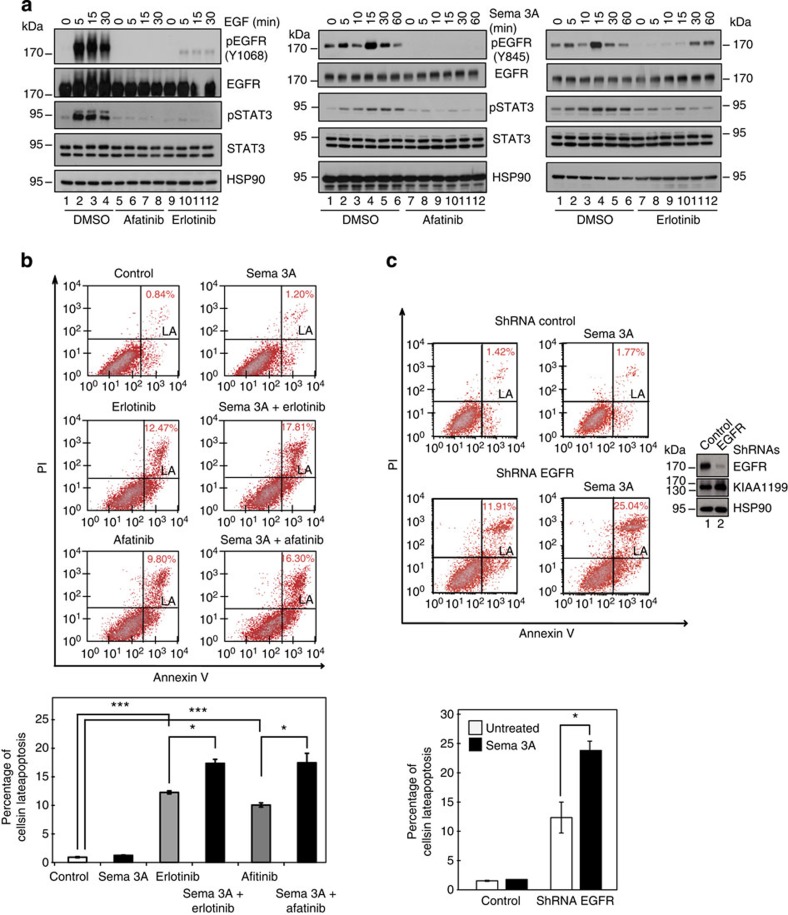
EGFR inhibition potentiates Semaphorin 3A-dependent cell death. (**a**) Pharmacological EGFR inhibitors interfere with EGFR phosphorylation upon EGF or Semaphorin 3A stimulation in cervical cancer cells. CaSki cells were pre-treated with dimethyl sulfoxide (DMSO) (vehicle as control) or with Afitinib or Erlotinib (1 μM) for 48 h (left and right panels). On the left, cells were subsequently serum-starved for 24 h and untreated or stimulated with EGF (100 ng ml^−1^) for the indicated periods of time. On the right, cells were untreated or stimulated with Semaphorin 3A (100 ng ml^−1^), as indicated. The resulting cell extracts were subjected to WB analyses. (**b**) Enhanced cell death on Semaphorin 3A stimulation in CaSki cells treated with EGFR inhibitors. FACS analysis were conducted on cells treated for 48 h with DMSO (vehicle as control) or with Erlotinib (1 μM), Afitinib (1 μM), Semaphorin 3A (100 ng ml^−1^) or with both Semaphorin 3A and Erlotinib or Afitinib, as indicated. Cells in late apoptosis (LA) were quantified. The histogram shows data obtained from one experiment done in quadruplicates. Error bars denote s.d. (Student’s *t*-test, *P*-values: ***<0.001; *P*-values: *<0.05). (**c**) Enhanced cell death on Semaphorin 3A stimulation in EGFR-depleted CaSki cells. FACS analysis were conducted on control or EGFR-depleted CaSki cells left untreated or stimulated for 48 h with Semaphorin 3A (100 ng ml^−1^), as indicated. Cells in late apoptosis (LA) were quantified. The histogram shows data obtained from three independent experiments done in triplicates. Error bars denote s.d. (Student’s *t*-test, *P*-values: *<0.05).

As Semaphorin 3A triggers cell death only on KIAA1199 deficiency and given the fact that EGFR levels are decreased in KIAA1199-depleted cells, we hypothesized that Semaphorin 3A-mediated cell death is negatively regulated by EGFR signalling. To explore this issue, we addressed Semaphorin 3A-mediated cell death in circumstances in which EGFR was pharmacologically blocked by Afatinib or Erlotinib treatments. Afatinib or Erlotinib efficiently inhibited EGF- and Semaphorin 3A-dependent EGFR phosphorylations in CaSki cells (Fig. 6a). As expected, Semaphorin 3A failed to trigger cell death in control cells 48 h post stimulation (from 0.84% to 1.20%) (Fig. 6b). Moreover, Afatinib or Erlotinib triggered cell death in CaSki cells (from 0.84% to 12.47% and from 0.84% to 9.80%, respectively), confirming that EGFR signalling is critical for cell survival in cervical cancer cells. Interestingly, as Semaphorin 3A stimulation further increased the number of cells in late apoptosis on EGFR inhibition with Erlotinib or Afatinib (from 12.47% to 17.81% or from 9.80% to 16.30%, respectively), an additional Semaphorin 3A-dependent pathway distinct from the one triggered on EGFR inhibition also contributes to cell death (Fig. 6b). Consistently, EGFR depletion in CaSki cells also triggered cell death as up to 11.91% of cells were in late apoptosis (Fig. 6c). Importantly, cell death was even more dramatic on Semaphorin 3A stimulation in EGFR-depleted cells (from 11.91% to 25.04%), which further supports the notion that Semaphorin 3A can trigger a pro-cell death pathway, but only in circumstances in which EGFR activity is inhibited (Fig. 6c). Taken together, our data indicate that KIAA1199 counteracts Semaphorin 3A- and Plexin A2-mediated cell death, mainly (but not exclusively) through EGFR phosphorylation.

As KIAA1199 stabilizes EGFR and because EGFR signalling limits TNFα-mediated apoptosis^49^, we next assessed cell death on TNFα stimulation in control versus KIAA1199-deficient CaSki cells (Supplementary Fig. 8). The TNFα-dependent apoptosis was much more dramatic on KIAA1199 deficiency (4.83% and 48.47% were in late apoptosis in control versus KIAA1199-depleted cells, respectively). Therefore, KIAA1199 limits cell death triggered by Semaphorin 3A or by TNFα, two pathways negatively regulated by EGFR signalling.

### KIAA1199 promotes EGFR signalling

**Figure 7:**
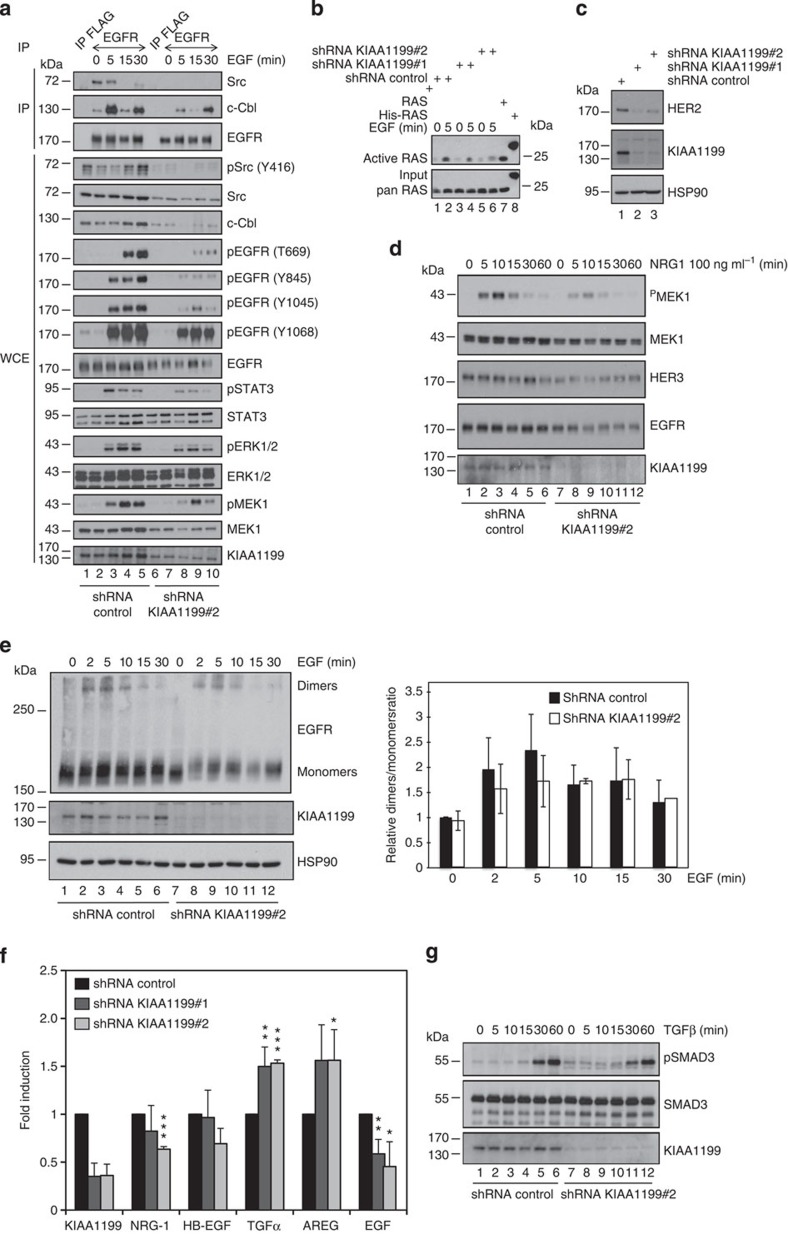
KIAA1199 promotes EGF-dependent signalling. (**a**) Defective EGF-dependent signalling on KIAA1199 deficiency in cervical cancer cells. Serum-starved control or KIAA1199-depleted CaSki cells (shRNA KIAA1199#2) were untreated or stimulated with EGF (100 ng ml^−1^) from 5 to 30 min. The resulting cell extracts were subjected to anti-FLAG (negative control) or -EGFR IPs followed by anti-Src, -c-Cbl and -EGFR WBs (top three panels). Crude cell extracts were also subjected to WB analyses using the indicated antibodies. (**b**) Optimal EGF-dependent Ras activation requires KIAA1199 in CaSki cells. Ras activity on stimulation with EGF (10 ng ml^−1^) was assessed in control or KIAA1199-depleted cells. Ras^+^ and His-Ras were used as positive controls. An anti-pan-RAS WB was carried out for normalization purposes (‘input’, bottom panel). (**c**) Defective HER2 expression on KIAA1199 deficiency in cervical cancer cells. Extracts from control or KIAA1199-depleted CaSki cells were subjected to WB analyses using the indicated antibodies. (**d**) Defective MEK1 activation on stimulation by NRG-1 in KIAA1199-deficient cervical cancer cells. Serum-starved control or KIAA1199-deficient CaSki cells were untreated or stimulated with NRG1 (100 ng ml^−1^) for the indicated periods of time and the resulting cell extracts were subjected to WB analyses, using the indicated antibodies. (**e**) KIAA1199 is dispensable for EGFR dimer formation on EGF stimulation in cervical cancer-derived cells. Serum-starved CaSki cells were unstimulated or treated with EGF (100 ng ml^−1^) for the indicated periods of time. Cells were subsequently cross-linked using 1 mM of bis(sulfosuccinimidyl) suberate (BS^3^) as chemical cross-linker. On the left, extracts were subjected to anti-EGFR WB analyses to detect both EGFR monomers and dimers. On the right, quantification of the dimer/monomer/HSP90 ratio in both control and KIAA1199-depleted cells subjected to EGF stimulations or not. The value of the dimer/monomer/HSP90 ratio in control and unstimulated cells was set to 1 and values obtained in other experimental conditions were relative to that. Data from three independent experiments (means±s.d.) are shown. (**f**) KIAA1199 differentially controls the expression of ErbB ligands in CaSki cells. Total RNAs from control or KIAA1199-depleted (shRNA KIAA1199#1 or shRNA KIAA1199#2) CaSki cells were subjected to real-time PCR to assess mRNA levels of the indicated ErbB ligands. The abundance of transcripts in control cells was set to 1 and their levels in KIAA1199-depleted cells were relative to that after normalization with glyceraldehyde 3-phosphate dehydrogenase (GAPDH). Data from three independent experiments performed in triplicates (means±s.d.) are shown (Student’s *t*-test, *P*-values: ***<0.001; *P*-values: **<0.01; *P*-values: *<0.05). (**g**) KIAA1199 is dispensable for TGFβ-dependent SMAD3 phosphorylation in cervical cancer cells. Control or KIAA1199-depleted CaSki cells were untreated or stimulated with TGFβ (5 ng ml^−1^) for the indicated periods of time and WB analyses were carried out on the resulting cell extracts.

Having defined KIAA1199 as an EGFR-binding protein, we next investigated whether KIAA1199 promotes EGF-dependent EGFR signalling. EGFR phosphorylations on all tested residues (T669, Y845, Y1045 and Y1068) were defective in KIAA1199-deficient CaSki cells (Fig. 7a). Moreover, Src and c-Cbl levels were severely downregulated on KIAA1199 deficiency. As a result, their recruitment to EGFR was strongly impaired (Fig. 7a). Consistent with a key role of KIAA1199 in EGFR signalling, EGF-dependent and Raf-mediated MEK1 phosphorylation on S217/221 was also defective in KIAA1199-deficient cells, most probably due to decreased MEK1 levels in those cells. Finally, EGF-mediated ERK1/2 and STAT3 phosphorylations were also critically relying on KIAA1199 in CaSki cells (Fig. 7a). Moreover, EGF-mediated Ras activation was also impaired on KIAA1199 depletion in these cells (Fig. 7b). KIAA1199 does not exclusively stabilize EGFR levels as HER2 and HER3 protein levels were also decreased in KIAA1199-depleted CaSki cells (Fig. 7c,d). Of note, KIAA1199 overexpression alone in SiHa cells modestly potentiated EGF-dependent ERK1/2 phosphorylations but did not enhance EGFR-, Src- and MEK1-phosphorylated levels (Supplementary Fig. 9), most probably because low endogenous KIAA1199 levels in those cells are sufficient to assemble an active EGFR signalosome.

To support the notion that KIAA1199 transmits signals from other ErbB members, we noticed that Neuregulin-1 (NRG-1), which mainly (but not exclusively) triggers signalling pathways through HER2 and HER3, failed to properly activate MEK1 in KIAA1199-depleted CasKi cells (Fig. 7d). Therefore, ErbB members rely on KIAA1199 to signal in cervical cancer-derived cells. As EGFR phosphorylations on multiple residues were impaired on KIAA1199 deficiency, we next addressed whether KIAA1199 was required for EGFR dimerization. Unstimulated or EGF-treated control and KIAA1199-deficient CaSki cells were chemically cross-linked and extracts were subjected to western blot analyses to detect both EGFR monomers and dimers. As expected, EGFR dimers were detected on EGF stimulation in control cells (Fig. 7e). Although less dimers were visualized in KIAA1199-depleted cells, EGFR monomers were also less detected in those cells (Fig. 7e). As a result, the EGFR dimer/monomer ratio did not change on KIAA1199 deficiency, suggesting that KIAA1199 is not required for EGFR dimerization on signalling.

We next wondered whether KIAA1199 was required for the expression of ErbB ligands. KIAA1199 may indeed sustain an autocrine EGF-dependent EGFR signalling pathway in transformed keratinocytes as EGF, but not NRG-1 or HB-EGF mRNA levels were decreased on KIAA1199 deficiency in CaSki cells (Fig. 7f). Of note, transforming growth factor (TGF)-α and AREG mRNA levels were paradoxically increased in KIAA1199-deficient CaSki cells, suggesting that KIAA1199 differentially controls the expression of ErbB ligands (Fig. 7f). Importantly, KIAA1199 specifically transmits signals from ErbB members but not from other receptors as KIAA1199 was dispensable for SMAD3 phosphorylation on TGFβ stimulation (Fig. 7g).

**Figure 8:**
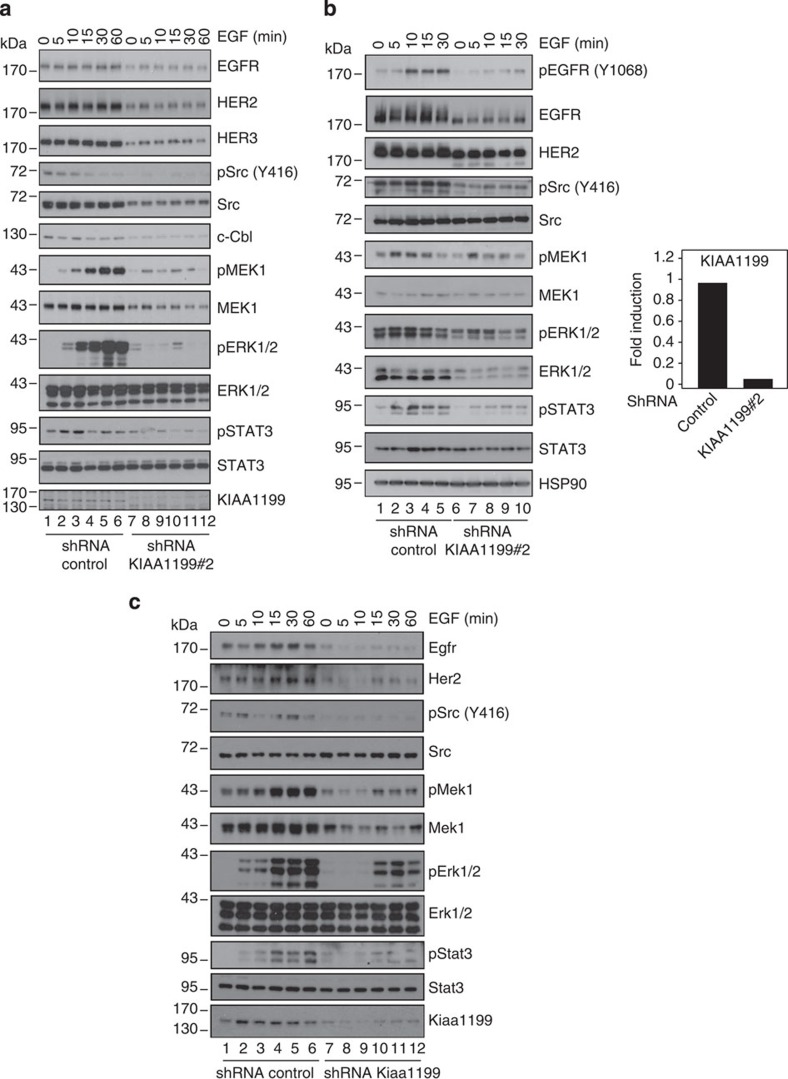
EGF-dependent signalling requires KIAA1199 in breast cancer-derived cells. (**a**,**b**) Impaired expression of ErbB members and defective EGFR signalling on KIAA1199 deficiency in breast cancer-derived cells. On the left, serum-starved control or KIAA1199-deficient MCF7 (**a**) or SK-BR-3 (**b**) cells were untreated or stimulated with EGF (100 ng ml^−1^) for the indicated periods of time and the resulting cell extracts were subjected to WB analyses using the indicated antibodies. On the right, total RNAs from control or KIAA1199-depleted (shRNA KIAA1199#2) CaSki cells were subjected to real-time PCR to assess KIAA1199 mRNA levels. The abundance of transcripts in control cells was set to 1 and their levels in KIAA1199-depleted cells were relative to that after normalization with glyceraldehyde 3-phosphate dehydrogenase (GAPDH). (**c**) KIAA1199 promotes EGF-dependent signalling in immortalized breast epithelial cells. Serum-starved control or KIAA1199-depleted NMuMG cells were serum starved and were untreated or stimulated with EGF (100 ng ml^−1^) for the indicated periods of time. WB analyses were conducted with the resulting cell extracts.

Given the decreased levels of Src, c-Cbl and MEK1 on KIAA1199 deficiency in CaSki cells, our data thus demonstrate that KIAA1199 is required for the integrity and activation of EGFR by connecting downstream kinases to this receptor. Importantly, this conclusion is not restricted to transformed keratinocytes, as EGF-mediated Src, -MEK1, -ERK1/2 and -STAT3 phosphorylations were also severely impaired on KIAA1199 deficiency in breast cancer-derived MCF7 and SK-BR-3 cells, as well as in immortalized breast epithelial NMuMG cells (Fig. 8a–c). EGFR, HER2 and HER3 protein levels were also decreased on KIAA1199 deficiency, as seen in CaSki cells (Fig. 8a–c). Therefore, KIAA1199 critically regulates the stability of ErbB members and thus promotes ErbB signalling in distinct cell types.

### KIAA1199 limits EGFR degradation through lysosomes

**Figure 9:**
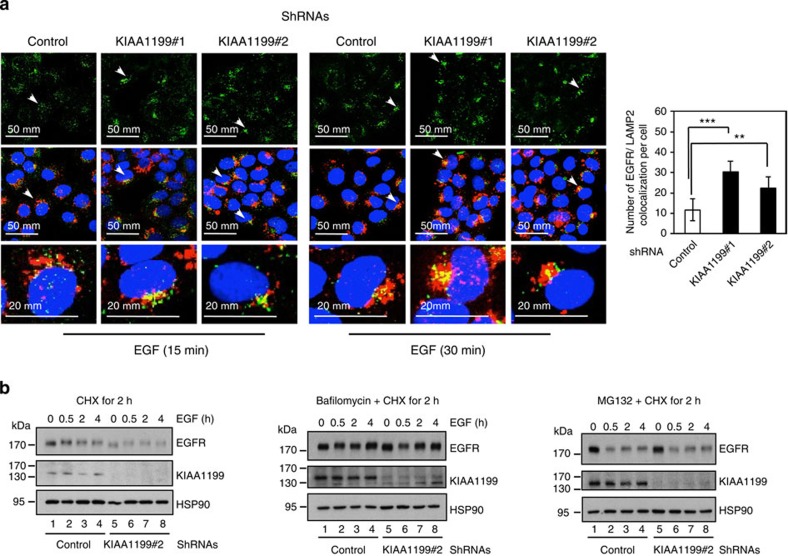
KIAA1199 limits EGF-dependent EGFR degradation through lysosomes. (**a**) KIAA1199 deficiency enhances EGFR co-localization with lysosomes on EGF stimulation. Control or KIAA1199-deficient CaSki cells were serum starved and subsequently untreated or stimulated with EGF for 15 or 30 min and subjected to immunofluorescence (IF) analyses. EGF-bound to EGFR as well as LAMP2-positive lysosomes were detected. Arrows indicates examples of EGF-bound EGFR that co-localizes with LAMP2-positive lysosomes. On the right, a quantification of co-localization of EGF-bound EGFR with LAMP2-positive lysosomes per cell on 15 min of EGF stimulation is illustrated. Values obtained in control cells was set to 1 and values obtained in other experimental conditions were relative to that. Data from one representative experiment performed in triplicates (means±s.d.) are shown (Student’s *t*-test, *P*-values: ***<0.001; *P*-values: **<0.01). (**b**) KIAA1199 limits EGF-dependent EGFR degradation in cervical cancer-derived cells. Serum-starved control or KIAA1199-depleted CaSki cells were pre-treated with cycloheximide (CHX, 50 μM) alone (left panels), with a combination of CHX and Bafilomycin (0.1 μM), or with both CHX and MG132 (25 μM) (middle and right panels, respectively). The resulting cells were untreated or stimulated with EGF (100 ng ml^−1^) for the indicated periods of time. Cell extracts were subjected to anti-EGFR, -KIAA1199 and -HSP90 WBs.

KIAA1199 critically promotes EGFR signalling in multiple cell types, yet it was unclear why EGFR levels were decreased on KIAA1199 deficiency. To address this issue, we assessed EGF-dependent EGFR trafficking in control versus KIAA1199-depleted CaSki cells. As expected, EGFR partially co-localized with LAMP2, a lysosomal marker, on 15 min of stimulation with EGF (Fig. 9a). Interestingly, EGFR more strongly co-localized with LAMP2 in KIAA1199-depleted cells after 15 min of EGF stimulation, indicating that KIAA1199 limits EGFR trafficking to lysosomes. Consistently, EGFR levels decreased more severely in KIAA1199-depleted versus control CaSki cells subjected to EGF stimulations, as judged by western blot analyses (Fig. 9b). This enhanced EGF-dependent EGFR degradation seen on KIAA1199 deficiency relies on lysosomes as a pre-treatment of control or KIAA1199-depleted cells with Bafilomycin led to similar EGFR protein levels in both experimental conditions (Fig. 9b). Therefore, KIAA1199 maintains EGFR protein levels by limiting its lysosome-dependent degradation. Of note, KIAA1199 also limits EGFR turnover as MG132, a proteasome inhibitor, restored EGFR levels in unstimulated and KIAA1199-depleted cells, while having no effect on EGF-dependent EGFR degradation (Fig. 9). Taken together, our data define KIAA1199 as a protein that stabilizes EGFR protein levels by limiting its turnover and its degradation through lysosomes on EGF stimulation.

### KIAA1199 promotes EGF-dependent EMT

**Figure 10:**
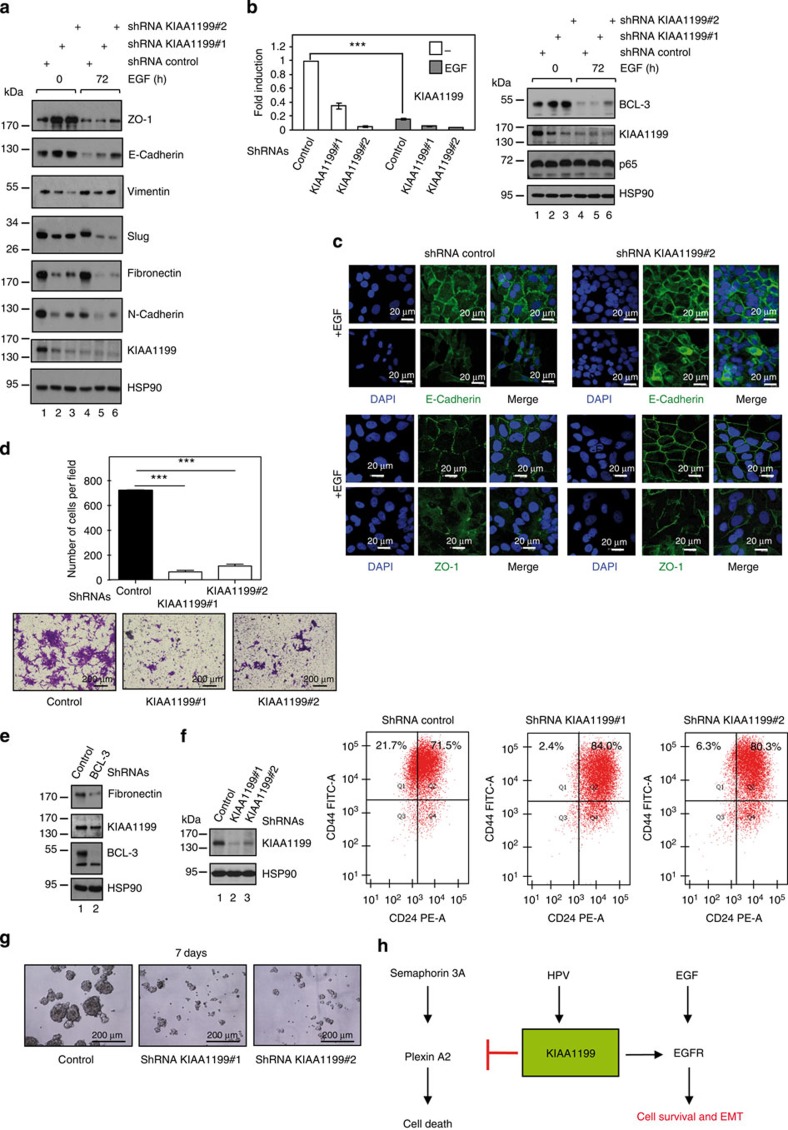
KIAA1199 promotes EGF-dependent EMT in cervical cancer cells. (**a**) Defective EGF-mediated EMT on KIAA1199 deficiency. Control or KIAA1199-depleted CaSki cells were untreated or stimulated with EGF (50 ng ml^−1^) for 3 days. The resulting cell extracts were subjected to WBs using the indicated antibodies. (**b**) EGF downregulates BCL-3 and KIAA1199, but not p65 expression. On the top, total RNAs from the six experimental conditions were subjected to TaqMan analyses to monitor KIAA1199 mRNA levels. The abundance of KIAA1199 transcripts in unstimulated control cells was set to 1 and their levels in other experimental conditions were relative to that after normalization with β-actin. Data from one representative experiment performed in triplicates (means±s.d., Student’s *t*-test, *P*-values: ***<0.001; *P*-values) are shown. On the right, cell extracts from the six experimental conditions were subjected to WBs. (**c**) EGF-mediated EMT is defective on KIAA1199 depletion in CaSki cells. Control versus KIAA1199-deficient CaSki cells were left untreated or stimulated with EGF for 72 h. IFs were subsequently conducted in the resulting cells to monitor E-cadherin and ZO-1 localizations/expressions. (**d**) Impaired invasiveness of KIAA1199-deficient cervical cancer cells. Control or KIAA1199-depleted CaSki cells were subjected to a chemotaxis assay using a Boyden chamber (see methods for details) to assess their invasive potential. Migrated cells were randomly photographed and counted in three fields per well. Quantifications were done based on three fields per experimental condition (means±s.d., Student’s *t*-test, *P*-values: ***<0.001). (**e**) BCL-3 deficiency in CaSki cells impairs both KIAA1199 and fibronectin expressions. CaSki cells were infected with the indicated lentiviral shRNA constructs. WBs on the resulting cell extracts are illustrated. (**f**) KIAA1199 deficiency impairs the pool of cancer stem cells (CD44^+^/CD24^−^ cells). Control or KIAA1199-deficient CaSki cells were subjected to FACS analyses to quantify the percentage of CD44^+^/CD24^−^ cells. (**g**) Impaired tumour sphere formation on KIAA1199 deficiency. Control or KIAA1199-deficient CaSki cells were cultured as spheres under stem cell conditions (see methods for details) and pictures were taken 7 days post plating. (**h**) Model illustrating KIAA1199 functions in cell survival and EMT. HPV induces KIAA1199 mRNA expression through BCL-3. KIAA1199 associates with EGFR to drive EGF-dependent Src, Ras, MEK1 and ERK1/2 activations, as well as EGF-mediated EMT. KIAA1199 is also a Plexin-A2-binding protein and protects cervical cancer cells from Semaphorin 3A-mediated cell death.

As EGF triggers EMT in CaSki cells^50^, we assessed protein levels of epithelial and mesenchymal markers in KIAA1199-deficient cells on EGF stimulation. Mesenchymal markers such as Slug, Fibronectin, Vimentin and N-cadherin decreased on KIAA1199 deficiency in both unstimulated and EGF-treated cells (Fig. 10a). Conversely, the expression of epithelial markers such as ZO-1 and E-Cadherin increased in KIAA1199-depleted CaSki cells (Fig. 10a). Of note, KIAA1199 protein and mRNA levels severely decreased after prolonged EGF treatment, similar to BCL-3 but not p65 (Fig. 10b). Therefore, our data strengthen the notion that BCL-3 critically drives KIAA1199 expression in CaSki cells. To further explore the role of KIAA1199 in EMT, we next performed immunofluorescence analyses and showed that KIAA1199 deficiency impaired re-localization of both E-cadherin and ZO-1 observed on EGF stimulation in CaSki cells (Fig. 10c). Therefore, KIAA1199 promotes EGF-induced EMT in cervical cancer cells. As EMT is linked to cell invasion, we next assessed the invasive potential of control and KIAA1199-deficient cells in a chemotaxis assay, using a Boyden chamber and a gradient of EGF. Although control CaSki cells efficiently migrated, KIAA1199 deficiency severely impaired cell invasiveness (Fig. 10d). BCL-3 deficiency in CaSki cells also deregulated Fibronectin expression (Fig. 10e). Thus, BCL-3 is required for EGF-dependent EMT and for cell invasion of cervical cancer cells, at least by promoting KIAA1199 expression.

As cells undergoing EMT acquire features of cancer stem cells^51^ and given the fact that KIAA1199 critically promotes EMT, we next assessed whether KIAA1199 deficiency impaired the pool of cancer stem cells, defined as CD44^+^/CD24^−^ cells. Although 21.7% of control CaSki cells were CD44^+^/CD24^−^, KIAA1199 deficiency dramatically decreased the pool of cancer stem cells as only 2.4% to 6.3% were CD44^+^/CD24^−^, depending on the efficiency of the shRNA-mediated depletion of KIAA1199 (Fig. 10f). Moreover, the number of spheres obtained with CaSki cells cultured under stem cell conditions dramatically relies on KIAA1199, as only control but not KIAA1199-depleted cells generated spheres of 100–400 μm in diameter 7 days post plating (Fig. 10g). Taken together, our data indicate that the pool of cancer stem cells critically relies on KIAA1199.

## Discussion

We describe here the characterization of KIAA1199, an oncogenic protein whose expression is induced by NF-κB and BCL-3 in transformed keratinocytes. KIAA1199 expression is positively correlated with HPV status and is aberrantly expressed in cervical (pre)neoplastic lesions. KIAA1199 acts as a molecular link between both Plexin A2 and EGFR-dependent cascades to protect from Semaphorin 3A-mediated cell death, at least by maintaining EGFR protein levels. KIAA1199 also promotes EGFR signalling by connecting EGFR to Src and downstream kinases. As such, KIAA1199 critically controls EGF-mediated EMT through the Ras/MEK1/ERK1/2 pathway (Fig. 10h).

The search for BCL-3-dependent genes has been challenging, as candidates are cell-type and context dependent. We demonstrated here that KIAA1199 is induced by BCL-3 in immortalized keratinocytes as well as in cervical cancer-derived cells. Both BCL-3 and KIAA1199 expressions similarly increased on PMA/TPA treatment in keratinocytes and also similarly decreased on prolonged EGF stimulation in CaSki cells. Those results strengthen the notion that BCL-3, as a p50- and p52-interacting protein, critically drives KIAA1199 expression in transformed keratinocytes. Although TNFα, which mainly transmits signals through the heterodimer p50/p65, does not induce KIAA1199 expression in HaCat, SiHa and CaSki cells, our data nevertheless showed that p65 also contributes to KIAA1199 expression in unstimulated CaSki and SiHa cells. Those data are in agreement with another study in which the capacity of overexpressed p65 to drive *KIAA1199* expression in COS cells was reported^52^. As TNFα failed to enhance KIAA1199 mRNA levels in the BCL-3-expressing CaSki cells, our data do not favour the hypothesis that BCL-3 synergizes with the canonical NF-κB-activating pathway to induce KIAA1199 gene expression. We rather favour a model in which KIAA1199 mRNA expression results from alternative collaborative effects between both BCL-3 and p65 in transformed keratinocytes. Molecular mechanisms underlying this phenomenon remain to be fully elucidated.

Although cross-talks between Plexins and RTKs were described in cancer cells^47^,^48^, molecular mechanisms by which Semaphorin receptors transactivate RTKs remained unclear. We showed here that Semaphorin 3A relies on KIAA1199 to transiently trigger EGFR phosphorylation in cervical cancer cells. As Plexin A2 deficiency enhanced Semaphorin 3A-dependent EGFR phosphorylations and also increased KIAA1199 protein levels, KIAA1199 acts as a pro-survival protein, at least by promoting EGFR signalling to limit Semaphorin 3A- and Plexin A2-mediated cell death in cervical cancer cells. Importantly, KIAA1199 also protects unstimulated cells from cell death, most probably by promoting an EGF-dependent autocrine EGFR signalling, given the fact that EGF mRNA levels were decreased on KIAA1199 deficiency in CaSki cells. Yet, mRNA levels of other EGFR ligands, namely TGFα and AREG, were negatively regulated by KIAA1199. Therefore, the autocrine EGFR signalling that relies on KIAA1199 is only relevant for EGF but not for other EGFR ligands.

We showed here that KIAA1199 protects from cell death triggered by Semaphorin 3A or by TNFα in cervical cancer cells. KIAA1199 limits Semaphorin 3A-dependent cell death by promoting EGFR signalling. This conclusion may also apply to TNFα, given the fact that EGFR activity is known to antagonize TNFα-dependent cell death^49^. Although our data suggest that KIAA1199 limits apoptosis, mainly by promoting EGFR signalling, we do not rule out the possibility that KIAA1199 also protects from cell death through EGFR-independent pathways. One may indeed expect KIAA1199 to directly interfere with pro-apoptotic, Plexin A2-dependent cascades as we demonstrated an additive effect between Semaphorin 3A and EGFR inhibition in triggering apoptosis in cervical cancer cells. How does Semaphorin 3A triggers cell death in circumstances in which EGFR signalling is blocked deserves to be further investigated. Of note, Plexin A2 deficiency severely but not completely abolished cell death triggered by Semaphorin 3A, which suggests that other pro-apoptotic pathways, potentially triggered through other Plexin members, may be counteracted by KIAA1199 expression.

Our data indicate that KIAA1199 is critical to assemble an active EGFR signalosome. Indeed, Src, c-Cbl and MEK1 protein integrities rely on KIAA1199 in cervical cancer cells, which suggests that KIAA1199, as an EGFR-binding protein, may provide a signalling platform to bring several downstream signalling proteins together to transmit pro-survival and invasive signals. Interfering with this signalling platform has dramatic consequences on EGF-mediated ERK1/2 and STAT3 activations, as well as on EGF-dependent EMT. However, it is noteworthy that KIAA1199 overexpression alone had modest consequences on EGF-dependent ERK1/2 phosphorylations, at least in SiHa cells, suggesting that the critical role of KIAA1199 in EGFR signalling can only be revealed through loss-of-function experiments (Supplementary Fig. 9). This conclusion applies to many scaffold proteins that lack, by definition, any catalytic activity. One example is NEMO/IKKγ, which acts in the canonical NF-κB-activating IKK complex^53^,^54^. Similar to KIAA1199 in EGFR signalling, overexpressed NEMO/IKKγ does not enhance NF-κB activation, yet NEMO/IKKγ deficiency has dramatic consequences on IKK stability and activity.

The mechanisms by which KIAA1199 is required in EGF-dependent EGFR phosphorylations on multiple residues remain unclear. Our data indicate that KIAA1199 is dispensable for EGFR dimer formation but nevertheless sustains EGFR signalling by promoting EGFR phosphorylation by Src on Tyr 845, as well as phosphorylation on multiple other residues. We also show that KIAA1199 actually limits EGFR degradation through lysosomes on EGF stimulation and also limits EGFR turnover through the proteasome. However, it is noteworthy that EGFR phosphorylation on Tyrosine 1045, which negatively regulates EGFR levels by triggering its binding to c-Cbl for subsequent degradation in lysosomes, is defective on KIAA1199 deficiency. Moreover, c-Cbl is destabilized and its recruitment on EGFR is consequently impaired in KIAA1199-deficient cells. Therefore, the precise mechanisms by which KIAA1199 prevents excessive EGFR degradation through lysosomes and/or promotes EGFR recycling on EGF stimulation remains to be fully elucidated.

The essential role of KIAA1199 in EGFR signalling is not restricted to cervical cancer cells as similar observations were also made in all breast cancer cell lines tested so far. Moreover, KIAA1199 deficiency also impaired HER2 and HER3 protein levels in CaSki, SK-BR-3 and MCF7 cells, suggesting that all ErbB members potentially require KIAA1199 to activate downstream kinases. Future studies will be dedicated to the exhaustive characterization of all RTKs whose activation is KIAA1199-dependent.

NF-κB is required for the induction and maintenance of EMT in Ras-transformed epithelial cells^55^. As we demonstrated here that BCL-3-deficient CaSki cells have decreased expression of fibronectin and because mesenchymal markers are not properly expressed on KIAA1199 deficiency in cervical cancer cells, our data defined BCL-3 as an oncogenic protein that promotes EMT, at least by controlling KIAA1199 expression. As BCL-3 also directly drives N-cadherin expression in melanoma-derived cells^56^ and because KIAA1199-depleted CaSki cells had decreased N-cadherin expression despite intact BCL-3 protein levels, we conclude that BCL-3 controls the expression of mesenchymal markers through multiple mechanisms in transformed cells. Given the capacity of HPV16 E6/E7 proteins to trigger EMT through decreased E-cadherin expression in immortalized keratinocytes^57^,^58^, our data also suggest that KIAA1199, whose expression is correlated with HPV status, links E6 expression to EMT. As HPV16 E6 also prolongs EGFR signalling by enhancing internalization of its phosphorylated forms^59^, our data define KIAA1199 as a central factor connecting HPV16 E6 to EGFR signalling, EMT and cell invasion.

Addressing the biological roles of KIAA1199 may be relevant in non-cancerous lesions. Indeed, KIAA1199, whose expression is increased in synovial fibroblasts from patients with osteoarthritis or rheumatoid arthritis, is also involved in hyaluronan depolymerization^37^. Besides a role in sustaining EGFR signalling, KIAA1199 may thus also promote cell invasion through hyaluronan degradation.

Given the deregulated expression of KIAA1199 in multiple solid tumours^34^,^35^,^36^,^38^,^60^ and the essential role of EGFR-dependent pathways in tumour development and progression of a variety of epithelial malignancies, it is very much likely to be that KIAA1199 is a common rather than a lineage-specific oncogenic protein. As we show here that KIAA1199 and EGFR/HER2/HER3 levels are positively correlated in transformed keratinocytes and in breast cancer-derived cells, KIAA1199 may be essential for the constitutive activation of EGFR signalling seen in multiple malignancies. Moreover, as the pool of cancer stem cells also critically relies on KIAA1199, our data thus define KIAA1199 as a promising target to interfere with deregulated EGFR signalling in cancer.

## Methods

### Cell cultures and reagents

HEK 293, 293FT, Phoenix, HaCat, A431, C4-II, SiHa, EIL8, CaSki, MCF7, SK-BR-3 and NMuMG cells (all from ATCC/LGC Standards, Molsheim Cedex, France) were maintained in DMEM supplemented with 10% FCS, glutamine and antibiotics. EGF, NRG-1, TNFα, PMA/TPA, recombinant Human Semaphorin 3A Fc Chimera and TGFβ1 were from Roche Applied Science (Basel, Switzerland), Cell Signaling (Danvers, MA), Sigma-Aldrich (St-Louis, MO), Peprotech (Rocky Hill, NJ, USA) and R&D Systems (Minneapolis, MN, USA), respectively. Erlotinib and Afatinib were purchased from Selleckchem (Houston, TX, USA). Polyclonal anti-KIAA1199 antibodies were raised in rabbits and were directed against a peptide derived from the C-terminal part of the human or mouse antigen (Phoenix Pharmaceutical, Burlingame, CA, USA). Anti-BCL-3, -p65, -Plexin A2, -Hsp90, -MEK1, -ERK1/2, -Myc, -STAT3 and -IκBα antibodies were from Santa Cruz Biotechnology (Santa Cruz, CA). Anti-Slug, -pMEK1 (S217/221), -Src, -pScr (Y416), -c-Cbl, -pSTAT3, -pERK1/2, -EGFR, -HER2, -HER3 and -pEGFR (T669, Y845, Y1045 and Y1068) antibodies were from Cell Signaling. The mouse anti-pCTD 4H8 RNA polymerase II antibody was from Millipore (Billerica, MA, USA). The anti-EGFR antibody used for immunoprecipitations was from GeneTex (Irvine, CA, USA). The anti-α-tubulin and -FLAG antibodies were from Sigma-Aldrich. Anti-Fibronectin, -E- and N-cadherin, as well as ZO-1 antibodies, were from BD Biosciences (San Jose, CA, USA). The anti-Vimentin antibody was from Akris (Herford, Germany). For western blot analyses, working dilutions for all antibodies were 1/1,000, except for the anti-E-cadherin and -FLAG antibodies, which were 1/2,000 and 1/5,000, respectively. Uncropped scans of the most important western blottings are illustrated in Supplementary Figs 10–19. Anti-histone H3 and histone H3K9 antibodies used for ChIP assays, as well as the anti-LAMP2 antibody used in immunofluorescence analyses, were from Abcam (Cambridge, UK). Anti-human CD44 FITC (fluorescein isothiocyanate) and CD24 phycoerythrin antibodies used for FACS analyses were from BD Pharmingen (San Jose, CA, USA).

FLAG-BCL-3, pBabe BCL-3, BCL-3 LAV, BCL-3 MTS expression plasmids and the pLL3.7 BCL-3 lentiviral constructs were previously described^20^,^61^. The pLL3.7 BCL-3 ΔN construct as well as the pBabe BCL-3 ANK M1,2,3 and K13-K26R mutants were generated by PCR using the corresponding FLAG-expressing construct as template. The KIAA1199-FLAG construct was generated by subcloning the corresponding PCR-amplified complementary DNA from CaSki cells into a pIRES puromycin construct. All ΔN- and ΔC-KIAA1199 FLAG mutants were generated by PCR using the KIAA1199-FLAG construct as template. The Myc-tagged mouse Plexin A2 cDNA construct was provided by Drs F. Suto and A. Chédotal (CNRS, UMR7102, Université Pierre et Marie Curie-Paris, Paris, France). A Myc-tagged construct that expresses the cytoplasmic domain of Plexin A3 (amino acids 1,242 to 1,871) was generated by subcloning the corresponding cDNA sequence from the pDONR223-Plexin A3 construct (Addgene, Cambridge, MA, USA) into the pCMV-Myc plasmid. Both Myc-tagged cytoplasmic domains of Plexin A1 (amino acids 1,306 to 1,896) and A4 (amino acids 1,311 to 1,894) were subcloned into the pCMV-Myc construct using cDNAs from CaSki cells. The FLAG-E6 expression construct was generated by subcloning the corresponding PCR amplified cDNA from CaSki cells into the pcDNA3.1-FLAG vector (Invitrogen, Carlsbad, CA, USA).

### Cervical biopsy specimens and PCR analysis

One hundred twenty-seven paraffin-embedded cervical biopsy specimens including 15 normal HPV-negative exocervical tissues and 112 HPV-positive cervical lesions (19 CIN grade I (CIN I), 20 grade II (CIN II), 37 grade III (CIN III) and 36 SCCs) were retrieved from the Tumor Bank of University of Liege. An informed written consent was obtained from all subjects. The protocol was approved by the Ethics Committee of University Hospital of Liege. For PCR analysis of cervical specimens, DNAs were prepared with the QIAamp DNA mini kit (Qiagen, Valencia, CA) and tested for the presence of HPV DNA sequences using consensus primers (GP5+/GP6+) from the L1 region^62^,^63^,^64^. This technique was shown to be able to amplify a 150-bp-long fragment of the L1 open reading frame of about 20 genital HPV genotypes^64^. HPV16 detection was performed using PCR with type-specific primers (synthesized by Eurogentec, Liege, Belgium). The nucleotide sequences were as follows: HPV16 forward, 5′-CAGAACCATATGGCGACAGC-3′ and reverse, 5′-GTACATTTTCACCAACAGCA-3′ (ref. 65). The amplimer length was ∼89 bp. Standard PCR conditions were 1 min at 95 °C, 1 min at the annealing temperature and 2 min at 72 °C for 40 cycles. After PCR, 25 to 30 μl of the samples were loaded on an agarose gel.

### Human normal primary keratinocytes culture

Primary foreskin keratinocytes (HPV negative) were derived from newborn foreskin via trypsin digestion at 37 °C (ref. 66). Briefly, the biopsy samples were incubated for 5–10 min in pure penicillin/streptomycin and Fungizone (vol/vol) at room temperature. Samples were subsequently digested for 1 h at 37 °C in 10 ml of trypsin EDTA containing 10% of antibiotics. The medium was discarded and epithelium was scraped in a petri dish. The samples were then rinsed twice with 10 ml pure serum. Serum was collected in a tube and cells were recovered after centrifugation. The dissociated human foreskin epithelial cells were suspended in keratinocyte-serum free medium (Gibco, Life Technologies, Paisley, UK) supplemented with bovine pituitary extract (25 μg ml^−1^), human EGF (0.1 ng ml^−1^), gentamicin, fungizone and penicillin/streptomycin (all from Gibco). The culture medium was changed twice per week from 7 days after plating, when stable colonies appeared. This study protocol was approved by the Ethics Committee of University Hospital of Liege.

### Immunohistochemistry and immunostaining assessment

For immunohistochemical analysis, paraffin sections were deparaffinized, rehydrated in graded alcohols and antigen retrieval was performed using citrate buffer in a pressure cooker for 6 min. The home-made anti-KIAA1199 antibody (1:350) was used for the primary reaction. Immunoperoxidase staining was performed using the LSAB kit (Dako, Glostrup, Denmark), according to the supplier’s recommendations. Positive cells were visualized using a 3,3′-diaminobenzidine substrate and the sections were counterstained with haematoxylin.

The immunolabelled tissues were evaluated by using a semi-quantitative score of the intensity and extent of the staining according to an arbitrary scale. For staining intensity, 0 represented samples in which the immunoreactivity was undetectable, whereas 1, 2 and 3 denoted samples with a low, moderate and strong staining, respectively. For staining extent, 0, 1, 2 and 3 represented samples in which the immunoreactivity was detectable in <5%, 6–25%, 26–75% and >75% of the epithelial cells, respectively. To provide a global score for each case, the results obtained with the two scales were multiplied, yielding a single scale of 0, +1, +2, +3, +4, +6 and +9 (ref. 67).

### ChIP assays

Cells were fixed in formaldehyde 1% for 10 min at room temperature, quenched in 1.25 M glycine and lysed in SDS 1% lysis buffer. Lysates were sonicated 15 min using the Bioruptor (Diagenode) and used for immunoprecipitation. Extracts were pre-cleared by 1 h incubation with protein A or G/Herring sperm DNA/BSA, and immunoprecipitation was performed by incubating overnight at 4 °C with the relevant antibody, using IgG antibody as negative control, and then 1 h with protein A or G/Herring sperm DNA/BSA. Protein–DNA complexes were washed as per standard ChIP techniques. After elution, proteinase K treatment and reversal of cross-links, DNA fragments were analysed by real-time PCR with SYBRG Green detection. Input DNA was analysed simultaneously and used as normalization. For normalization of the RNAPII ChIPs, the signal obtained from a non-coding region was used to compensate for possible fluctuations arising during handling. For the histone-related ChIPs, acetyl-histone-specific ChIP values were normalized according to the total H3 signal (as detected with the C terminus-specific anti-histone H3 antibody). Primers used are listed in Supplementary Table 1.

### RNA interference and viral infections

siRNAs were individually transfected or as a pool of four distinct sequences. siRNAs targeting BCL-3 were from Dharmacon (Lafayette, CO, USA), while siRNAs targeting E6 (5′-GAGGUAUAUGACUUUGCUU-TT-3′; 5′-AGACAUCUGGACAAAAAGCTT-3′; 5′-GAAUGUGUGUACUGCAAGCTT-3′; 5′-ACAACAAACCGUUGUGUGAUUTT-3′) were from Eurogentec (Liege, Belgium), as were both siRNAs targeting p65 (5′-GGUGCAGAAAGAGGACAUU-3′ and 5′-GGACAUAUGAGACCUUCAA-3′). The control or shRNAs targeting human KIAA1199, Plexin A2, EGFR or BCL-3 were from Sigma-Aldrich. Retroviral infections of HaCat cells were carried out according to instructions provided in the the website www.stanford.edu/group/nolan/retroviral_systems/retsys.html. Briefly, ecotropic Phoenix cells (2 × 10^6^) were transfected using the calcium phosphate method, with 10 μg of retroviral plasmids (pBabe-puromycin), either control (pBabe empty) or a construct encoding wild-type BCL-3 or BCL-3 mutants. Forty-eight hours after transfection, supernatants from those infected cells were collected, filtered (0.2 μm) and added with polybrene (8 μg ml^−1^) to 4 × 10^5^ HaCat cells. Forty-eight hours post infection, HaCat cells were treated for 5 days with puromycin (2 μg ml^−1^). Overexpression of proteins in HaCat cells were carried out through lentiviral infections as well. 293FT cells (3 × 10^6^) (ATCC/LGC Standards) were transfected with 12 μg of pLL3.7 lentiviral plasmids, either with a control or with a construct encoding wild-type BCL-3 or BCL-3 mutants, 12 μg of R8.91 and 5 μg of VSVG plasmids, using the Mirus Bio’sTransIT-LT1 reagent (Mirus, Pittsburg, PA, USA). The supernatants of those infected cells were collected and filtered (0.2 μm) 48 h after transfection and added with polybrene (8 μg ml^−1^) to 4 × 10^5^ HaCat cells. This latter step was repeated once after 24 h. For lentivirus-mediated shRNA experiments in CaSki cells, 293FT cells (3 × 10^6^) were transfected with 12 μg of the ‘non-target’ lentiviral shRNA plasmid (used as negative control) or with the shRNA construct that targets KIAA1199, 12 μg of R8.91 and 5 μg of VSVG plasmid, using the Mirus Bio’sTransIT-LT1 reagent. The supernatants of those infected cells were collected and filtered (0.2 μm) 48 h after transfection and added with polybrene (8 μg ml^−1^) to 4 × 105 CaSki cells. This latter step was repeated once after 24 h. The isopropyl-β-D-thiogalactoside-inducible shRNA construct (Vector MISSION 1 × LacO Inducible) was from Sigma. The MISSION 1 × LacO Inducible Non-Target shRNA was used as negative control. To carry out inducible depletions, shRNA-infected CaSki cells were treated with isopropyl-β-D-thiogalactoside for 5 days. Cells were subsequently serum starved for 24 h before any treatment with EGF.

### Immunofluorescences and immunoprecipitations and Ras activation assays

CaSki cells were seeded on coverslips in six-well plates. CaSki cells were untreated or stimulated with EGF for 72 h and then fixed with paraformaldehyde 4% and preimmobilized with Triton X 0.3% for 10 min at room temperature. Cells were then incubated with primary antibodies (E-cadherin and ZO-1) for 2 h at room temperature followed by 45 min of incubation at room temperature with FITC-conjugated antibodies (Dako). Images were acquired with the confocal system of Leica SP5 inverted microscope (Leica Microsystems, Wetzlar, Germany).

For immunoprecipitations involving endogenous proteins, CaSki cells were washed with cold PBS and lysed in ice-cold lysis buffer: 25 mM Tris–HCl pH 7.4, 150 mM NaCl, 1 mM EDTA, 1% NP-40 plus Complete (Roche). Cell lysates were incubated overnight at 4 °C with the indicated antibodies followed by 1 h incubation with protein A-agarose or G-agarose conjugate (Santa Cruz Biotechnology). Immunoprecipitations with anti-FLAG, IgG or pre-immune serum were performed in parallel as negative controls. The resulting immunoprecipitates were washed five times with the lysis buffer and subjected to SDS–PAGE electrophoresis for western blot analyses. For immunoprecipitations involving overexpressed proteins, 293 cells (3 × 10^6^) were transfected with the indicated expression plasmids using the Mirus Bio’sTransIT-LT1 reagent (Mirus) according to the manufacturer’s protocol. Twenty-four hours after transfection, cells were washed with ice-cold PBS and lysed. Immunoprecipitations with anti-FLAG or -Myc beads were performed for 4–6 h at 4 °C and washed five times with lysis buffer. EGFR trafficking on EGF stimulation was assessed as described^68^. Briefly, control or KIAA1199-depleted CaSki cells were seeded on coverslips in six-well plates and serum-starved for 24 h. Cells were subsequently untreated or stimulated with EGF (Alexa Fluor 488 EGF complex, Life Technologies, Grand Island, NY). Cells were kept for 45 min at 4 °C in the dark, put back at 37 °C for 10 min, washed with some fresh medium to remove EGF and incubated again at 37 °C for 15 or 30 min. Cell were then fixed with paraformaldehyde 4% and pre-immobilized with Triton X 0.3% for 10 min at room temperature. They were subsequently incubated with an anti-LAMP2 antibody for 2 h at room temperature followed by 45 min of incubation at room temperature with an Alexa Fluor 594-conjugated antibody (Dako). Images were acquired with the confocal system of Leica SP5 inverted microscope.

Ras activity was assessed using the Ras Activation Assay Biochem kit, according to the manufacturer’s protocol (Cytoskeleton, Denver, CO, USA). Briefly, CaSki cells were serum-starved overnight and subsequently left untreated or stimulated with EGF (10 ng ml^−1^) for 5 min. Resulting cells were lysed with supplied lysis buffer and 300 μg total proteins were used for immunoprecipitation of active Ras protein (GTP-Ras) using Raf-RBD beads at 4 °C. GTP-Ras-bound beads were washed twice with supplied washing buffer. Twenty microlitres of Laemmli buffer was added before boiling the samples. Experimental samples and proper controls were loaded and run on a SDS–PAGE and activated Ras was detected by western blot analysis using the anti-Pan Ras antibody.

### Chemical cross-linking of EGFR in intact cells

The detection of EGFR dimers in EGF-treated CaSki cells was carried out using 5mM of bis(sulfosuccinimidyl) suberate (BS3) (Pierce) as chemical cross-linker^69^. Briefly, cells were left unstimulated or treated with EGF for up to 30minutes and incubated for 30 minutes at room temperature with 5mM bis(sulfosuccinimidyl) suberate dissolved in PBS. Tris-HCl pH 7.5 (20mM) was subsequently added for 15 minutes at room temperature to terminate the cross-linking reaction followed by washes with ice-cold PBS. Cells were then solubilized with a lysis buffer (1% Triton, X-100, 10% glycerol, 50mM Hepes pH7.4, 1mM Sodium orthovanadate, 10mg/ml leupeptin and 1mM phenylmethylsulfonyl fluoride) prior to centrifugation at 14,000 x g for 10 minutes at 4°C to remove Triton-insoluble material. Equivalent amounts of proteins were then resolved by SDS-PAGE followed by anti-EGFR western blot analyses.

### Proximal ligation assay

CaSki cells were plated in 35 mm cell culture dish with glass bottom and fixed in 4% formaldehyde. Localization of the endogenous Plexin A2/KIAA1199 complex was achieved using the Duolink Red kit (Sigma). The Duolink In Situ PLA Probe Anti-Mouse PLUS, Duolink In Situ PLA Probe anti-Rabbit MINUS and Duolink In Situ Detection Reagents Red were used for staining purposes, using the anti-Plexin A2 monoclonal antibody (MAB10421) (Millipore) and our home-made anti-KIAA1199 polyclonal antibody, according to the protocol provided by the manufacturer. DAPI (4,6-diamidino-2-phenylindole) stainings were carried out to visualize nuclei. Images were acquired with the confocal system of Leica SP5 inverted microscope (Leica Microsystems).

### Thymidine incorporation assays

Counted cells were seeded in triplicates in 96-well plates. 3H-thymidine (0.4 μCi) was subsequently added in each well and cells were incubated for 16–18 h at 37 °C. Cells were harvested and the incorporated radioactivity was quantified on the TopCount NXT v2.53 scintillation counter (Perkin Elmer, Downers Grove, IL, USA).

### TUNEL assays and FACS analyses

TUNEL assays were carried out using the ApoAlert DNA Fragmentation Assay Kit (Clontech). Control or KIAA1199-depleted CaSki cells were untreated or stimulated with Semaphorin 3A for 48 or 72 h. The Annexin-V-FLUOS Staining Kit from Roche was used for detection and quantification of apoptosis. Proliferation was assessed by fixing and labelling control or KIAA1199-depleted CaSki cells using the Click-iT EdU cell proliferation assay kit (Invitrogen); 5-bromodeoxyuridine was added 2 h before fixing. Percentage of cells in S phase was based on the amount of EdU-FITC-positive cells. 7-Aminoactinomycin D (Sigma) was used for DNA content.

For FACS analyses, control or KIAA1199-depleted CaSki were trypsinized, harvested, centrifuged at 200 *g* for 5 min, washed in PBS/1%BSA and incubated with antibodies for 40 min. Cells were subsequently washed with PBS/1%BSA, resuspended in PBS and subjected to FACS analysis.

### Tumour sphere cultures

The generation of spheroid cultures were carried out as previously described^70^. Briefly, CaSki Cells were plated in Costar^R^ ultra low attachment plates (Corning, Tewksbury, MA, USA) in a serum-free medium supplemented with 20 ng ml^−1^ of EGF, 20 ng ml^−1^ (Stem Cells Technologies, Grenoble, France) and 0.4% BSA at a density of 1 × 10^3^ cells per ml for up to 7 days). Fresh medium was added every 48 h and pictures were taken once the majority of spheres from control CaSki cells reached 100–400 μm in diameter.

### Chemotaxis assays

Control or KIAA1199-deficient Caski cells were seeded as 20,000 cells per well with an EGF (100 ng ml^−1^) gradient. Migrated cells were stained with crystal violet 72 h after seeding. Three fields per well were randomly photographed for each experimental condition.

### Microarray analysis and real-time PCRs

For transcriptomic experiments, total RNAs were extracted from the resulting HaCat cells cultured in triplicates, using the RNeasy Minit kit from Qiagen. The integrity of the RNAs from those six distinct experimental conditions was checked with the Agilent Bioanalyzer using the RNA 6,000 Nano kit (Agilent, Santa Clara, CA, USA). Biotin-labelled cRNA, subsequent hybridization on the GeneChip Human Genome U133A 2.0 (Affymetrix, Santa Clara, CA) and scanning were performed following the manufacturer’s instructions. For real-time PCR analysis, total RNAs were extracted using the EZNA Total RNA kit (Omega Bio-Tek, Norcross, GA, USA) and cDNAs were synthesized using the Revert Aid H Minus First Strand cDNA Synthesis kit (Fermentas, Glen Burnie, MD, USA). Subsequent PCRs were carried out using the Power SYBR Green PCR Master kit (Applied Biosystems, Foster City, CA, USA) on the LightCycler 480 (Roche Applied Sciences). The primers to amplify β-actin or glyceraldehyde 3-phosphate dehydrogenase sequences were used for normalization purposes. Primer sequences are listed in Supplementary Fig. 2.

## Author contributions

K.S., X.Z., P.H., S.I.G., Z.J., I.K., J.H., P.R., B.H., A.L., J.S. and P.C. carried out the experiments. A.G. and P.D. provided reagents tools and human samples, respectively. K.S. and A.C. conceived the study, designed the experiments, analysed the data and wrote the manuscript.

## Additional information

**Accession codes:** Microarray data were deposited to the GEO database. The accession number is GSE60551.

**How to cite this article:** Shostak, K. *et al.* NF-κB-induced KIAA1199 promotes survival through EGFR signalling. *Nat. Commun.* 5:5232 doi: 10.1038/ncomms6232 (2014).

## Supplementary Material

Supplementary Information Supplementary Figures 1-19, Supplementary Tables 1-2
