# Supplementary material for: NF-κB-induced KIAA1199 promotes survival through EGFR signalling
Source: Nat Commun. 2014 Nov 4;5:5232. doi: 10.1038/ncomms6232 (PMC4241993; doi:10.1038/ncomms6232)
Supplement: Supplementary Information — Supplementary Figures 1-19, Supplementary Tables 1-2 [file ncomms6232-s1.pdf]

SUPPLEMENTARY INFORMATION

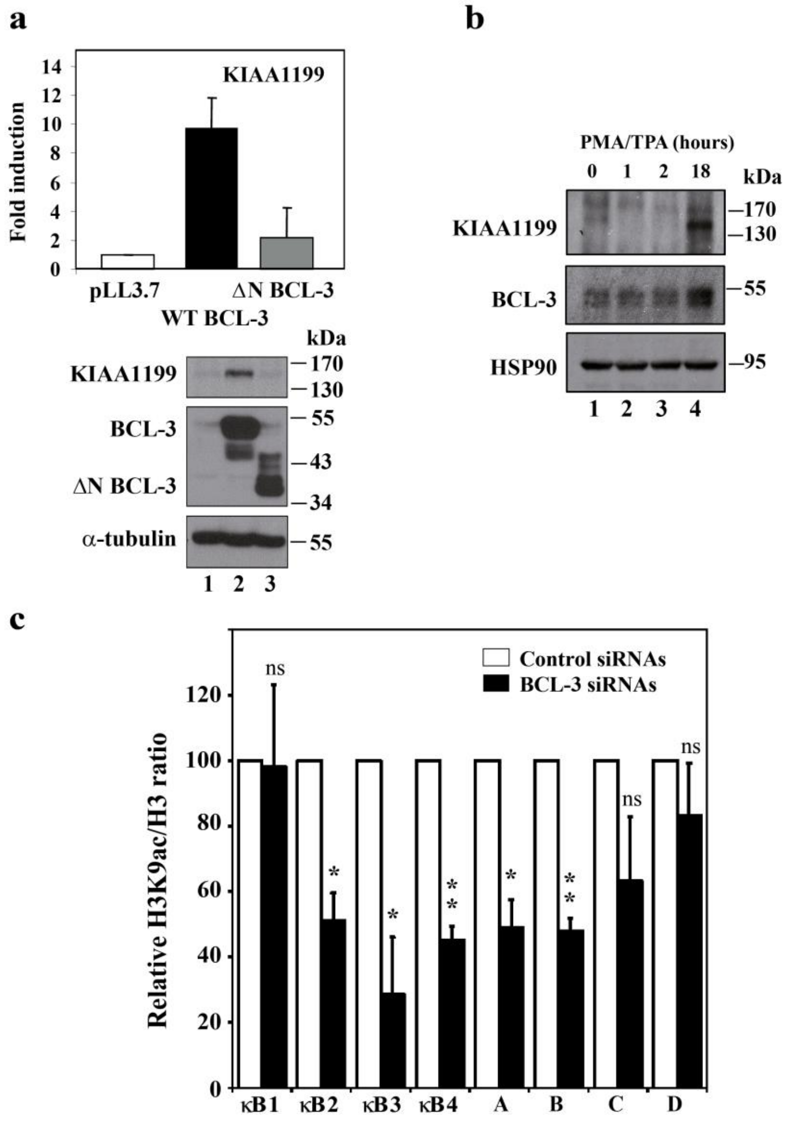

Supplementary Figure 1: BCL-3 promotes KIAA1199 expression in cervical cancer-derived cells. **A**. BCL-3 integrity is required to induce *KIAA1199* expression. HaCat cells were infected with a control lentivirus (pLL3.7) or with a lentiviral construct expressing WT BCL-3 or the ΔNBCL-3 mutant. On the top, elevated mRNA levels of KIAA1199 in cells upon BCL-3 overexpression. KIAA1199 mRNA levels in cells infected with the control lentivirus was set to 1 and levels in other experimental conditions were relative to that after normalization with 18S rRNA. Data from three independent experiments (means ± standard deviations) are shown. At the bottom, Western blots (WBs) performed on extracts from control, ΔNBCL-3- or BCL-3-overexpressing cells. **B**. KIAA1199 expression is TPA-inducible. HaCat cells were untreated or not with PMA/TPA (75nM) and WB analyses were done on SDS extracts. **C**. Impaired histone acetylation on the *KIAA1199* promoter in BCL-3 deficient CaSki cells. ChIP assays with an anti-histone H3K9ac antibody were done. Acetyl-histone values were normalized according to the total H3 signal (as detected with the anti-histone H3 antibody). Density in control cells was set to 100 and the values obtained in depleted cells expressed relative to that. Error bars denote standard deviation (Student t-test p-values: ***< 0.001; **<0.01; *< 0.05). ns= no significant.

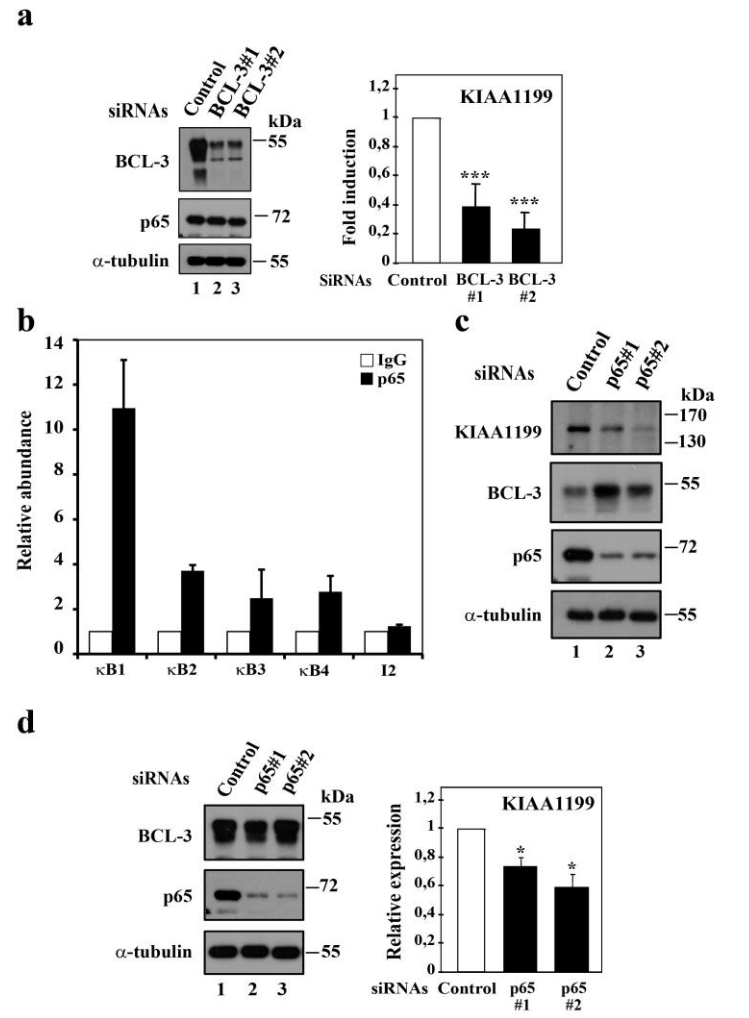

Supplementary Figure 2: BCL-3 and p65 control KIAA1199 expression in cervical cancer-derived cells. **A**. BCL-3 drives KIAA1199 expression. SiHa cells were transfected with the indicated siRNAs and cell extracts were subjected to WB analyses. Real-Time PCR to assess KIAA1199 mRNA levels were also carried out (right panel). KIAA1199 mRNA levels in SiHa cells transfected with the control siRNA ("GFP") was set to 1 and levels in other experimental conditions were relative to that after normalization with GAPDH. Error bars denote standard deviation (Student t-test p-values: ***< 0.001). **B**. Recruitment of endogenous p65 to the *KIAA1199* promoter. ChIP assays were done with an anti-p65 or -IgG (negative control) antibody and extracts from CaSki cells. Associated DNA was analysed by Real-Time PCR using primers spanning the κB sites. p65 density on every κB sites as well as on intron 2 ("I2") was relative to signals obtained with the anti-IgG antibody. Data obtained from one ChIP assay performed in triplicates are shown. Error bars denote standard deviation. **C**. KIAA1199 expression relies on p65. CaSki cells were transfected with the indicated siRNAs and cells extracts were subjected to WB analyses. **D**. p65 drives KIAA1199 expression. SiHa cells were transfected with the indicated siRNAs and cell extracts were subjected to WB analyses. Real-Time PCR to assess KIAA1199 mRNA levels were carried out (right panel). KIAA1199 mRNA levels in SiHa cells transfected with the control siRNA ("GFP") was set to 1 and levels in other experimental conditions were relative to that after normalization with GAPDH. Error bars denote standard deviation (Student t-test p-values: *< 0.05).

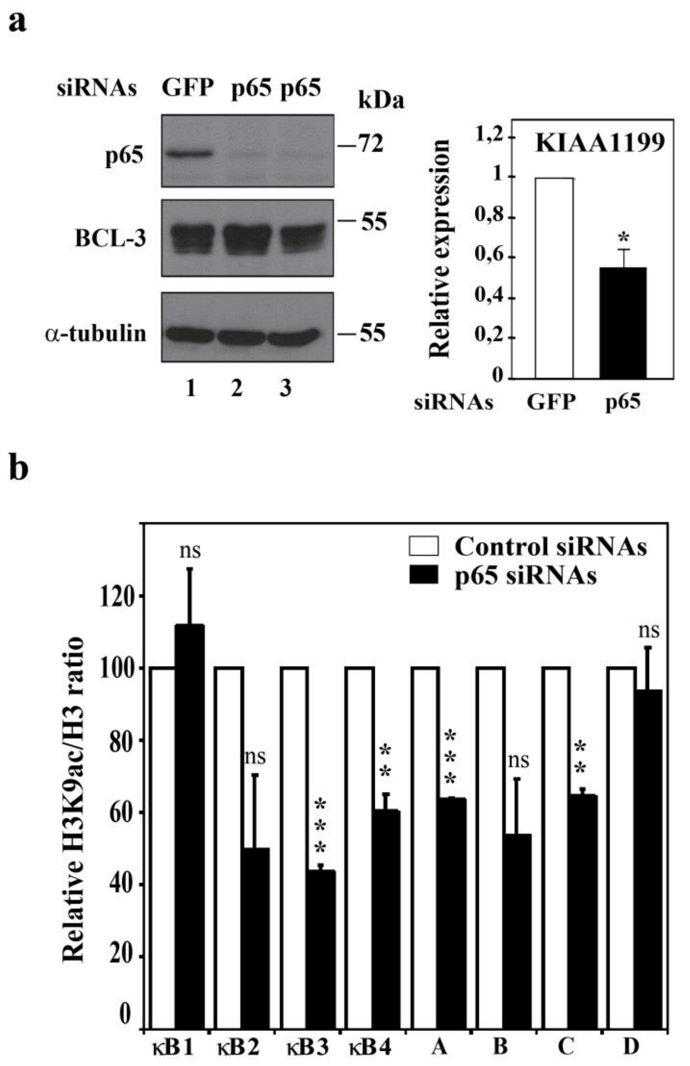

Supplementary Figure 3: p65 drives KIAA1199 expression. A. p65 depletion in BCL-3-overexpressing cells impairs KIAA1199 expression. BCL-3-overexpressing HaCat cells were transfected with a control siRNA ("GFP", lane 1) or with a siRNA targeting p65 (duplicates are shown, lanes 2 and 3). On the left, the resulting cells were subjected to WB analyses. On the right, KIAA1199 mRNA levels were assessed in the resulting cells. KIAA1199 transcripts in cells transfected with the control siRNA ("GFP") was set to 1 and their levels in cells transfected with other siRNAs were relative to that after normalization with 18S rRNA. Data from 3 independent experiments (means $\pm$ standard deviations) are shown (Student t-test p-values: *< 0.05). **B**. Impaired histone acetylation on the *KIAA1199* promoter upon p65 depletion. ChIP assays with an anti-histone H3K9ac-specific antibody were performed with control or p65-depleted CaSki cells. Acetyl-histone specific values were normalized according to the total H3 signal (as detected with the anti-histone H3 antibody). Density in control cells was set to 100 and the values obtained in depleted cells expressed relative to that. Error bars denote standard deviation (T-test p-values: ***< 0.001; **<0.01). ns= no significant.

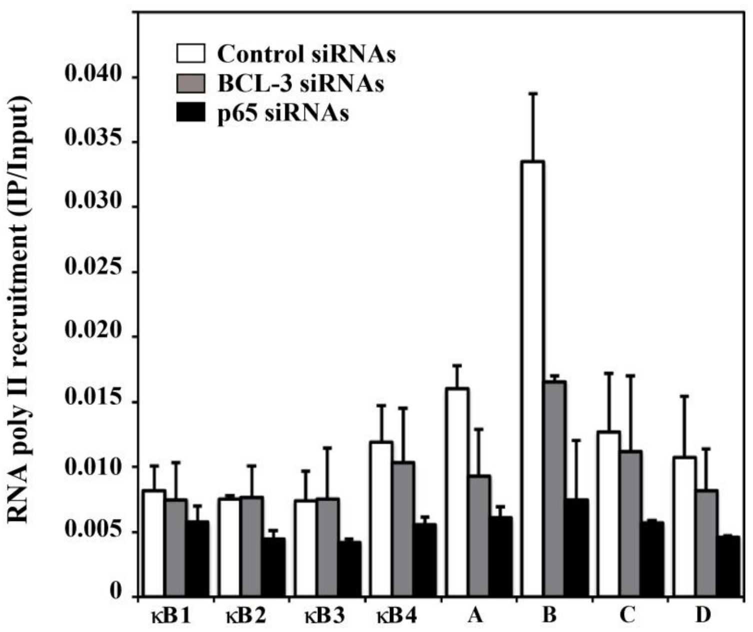

Supplementary Figure 4: Defective recruitment of RNA polymerase II on the *KIAA1199* promoter upon BCL-3 or p65 deficiency in CaSki cells. The recruitment of the activated form of RNA polymerase II was assessed by ChIP assays. ChIP Signals were expressed as log2 ratios IP/INPUT for each primer pair used. Error bars denote standard deviation. (Student t-test p-values: *< 0.05).

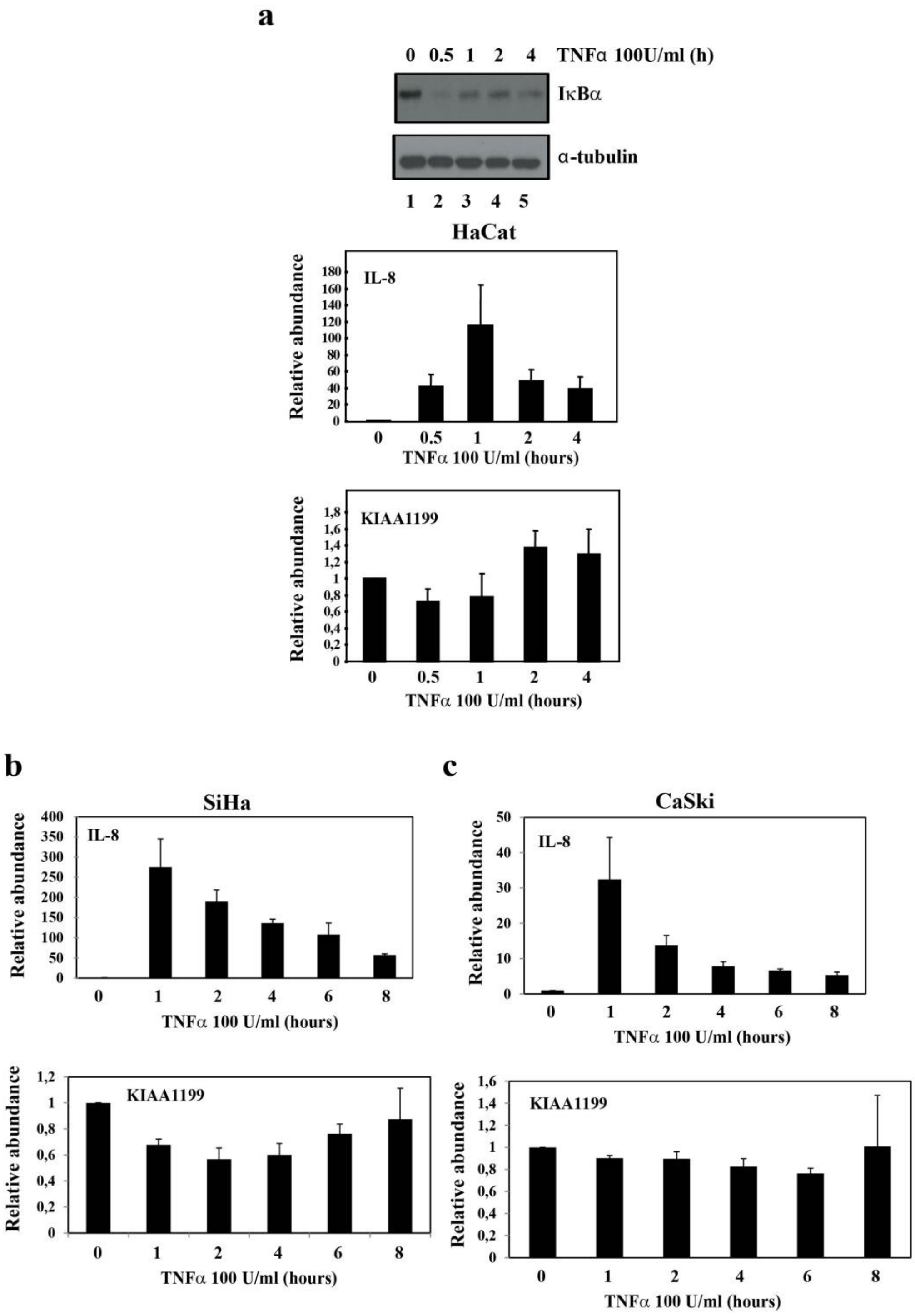

Supplementary Figure 5: IL-8 but not KIAA1199 is a TNFα-inducible gene. HaCat (A), SiHa (B) or CaSki (C) cells were left untreated or stimulated with TNFα for the indicated periods to time. On the top (A), the resulting protein cell extracts from HaCat cells were subjected to anti-IκBα (marker of NF-κB activation) and α-tubulin (loading control) WB analyses, as indicated. At the bottom, Real-Time PCR analyses were conducted to assess IL-8 and KIAA1199 mRNA levels. The abundance of transcripts in unstimulated cells was set to 1 and their levels in TNFα-stimulated cells were relative to that after normalization with 18S rRNA. Data from three (A) or two (B and C panels) independent experiments performed in triplicates (means ± standard deviations) are shown.

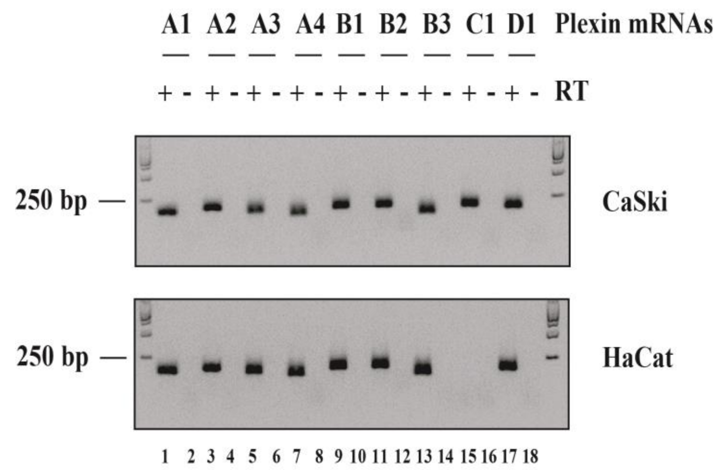

Supplementary Figure 6: Expression profile of Plexin members in CaSki and HaCat cells. RT-PCR experiments were conducted from total RNAs extracted from the indicated cell lines. The resulting PCR fragments were run on an agarose gel and stained. RT= reverse transcriptase.

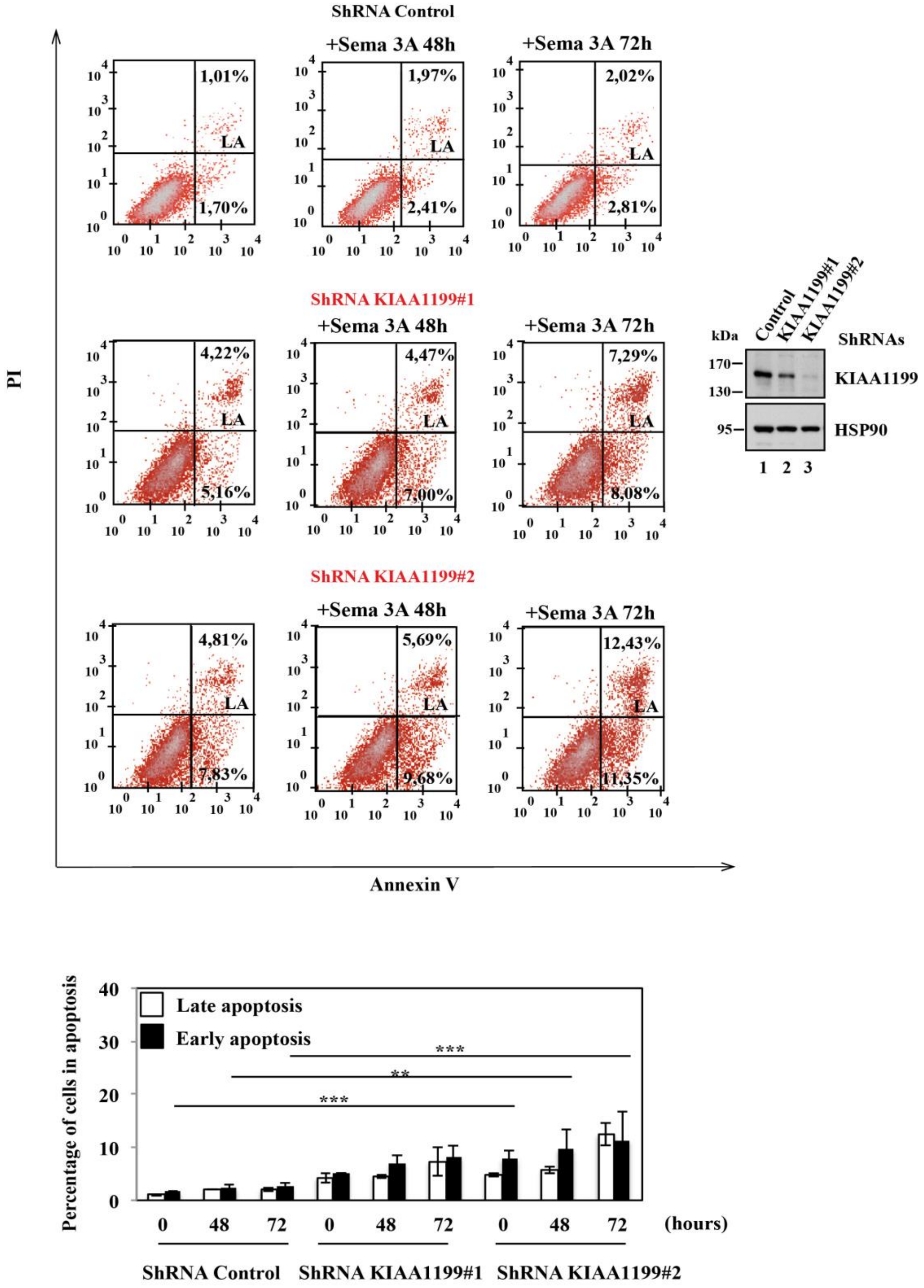

Supplementary Figure 7: KIAA1199 depletion sensitizes CaSki cells to Semaphorin 3A-mediated cell death. On the top, control or KIAA1199-depleted (shRNA KIAA1199#1 and shRNA KIAA1199#2) CaSki cells were untreated or stimulated with Semaphorin 3A (100 ng/ml) for 48 or 72 hours and FACS analyses were conducted to quantify early or late apoptotic (LA) cells. Anti-KIAA1199 and -HSP90 (loading control) WB analyses are also illustrated. At the bottom, FACS data from three independent experiments are illustrated in the histogram. Statistical analyses were done for control versus KIAA1199-depleted cells (shRNA KIAA1199#2) in early apoptosis (means ± standard deviations, Student t-test p-values: ***< 0.001; **<0.01).

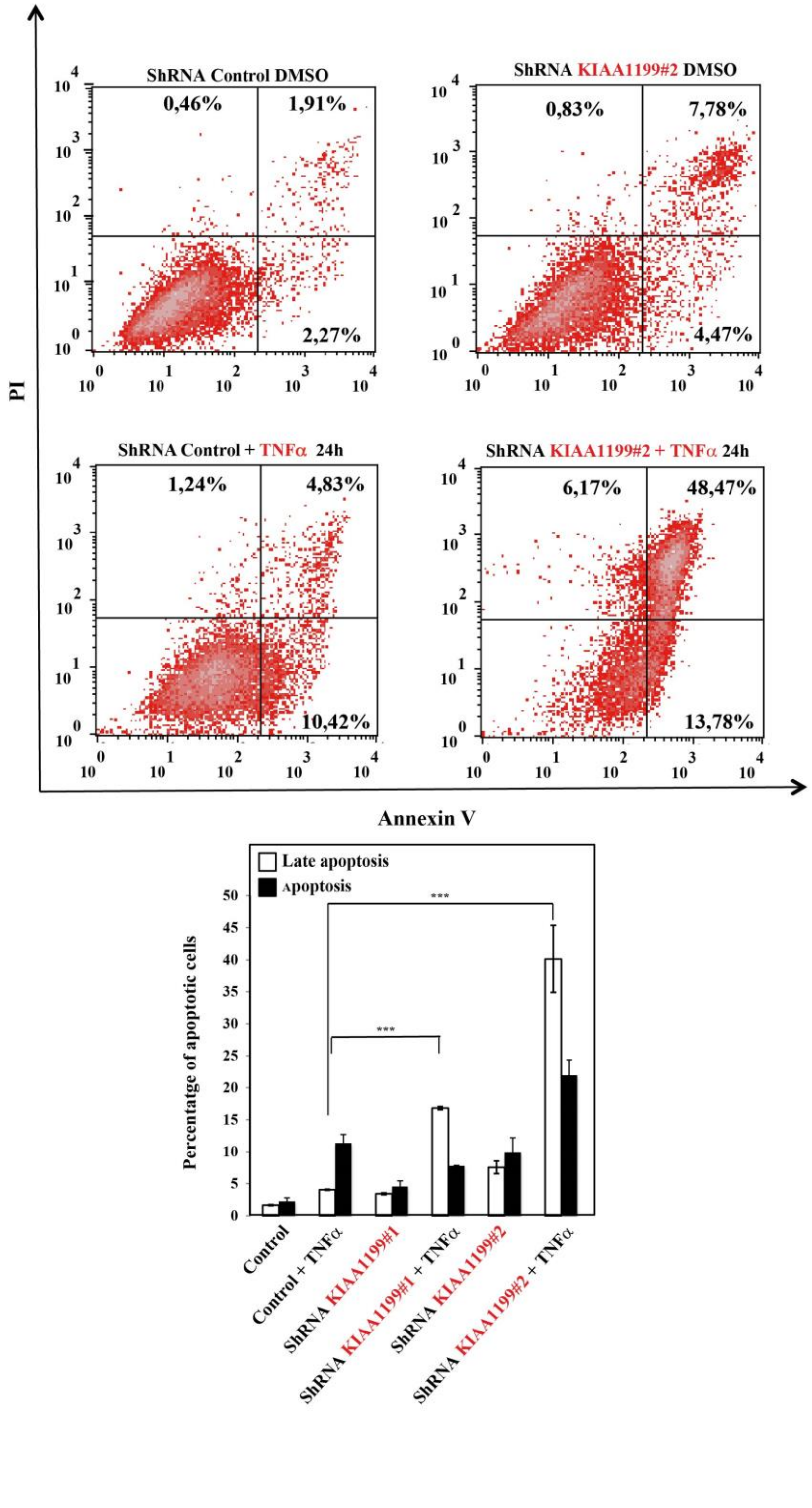

Supplementary Figure 8: KIAA1199 protects from TNFα-dependent cell apoptosis in cervical cancer-derived cells. Control or KIAA1199-depleted (shRNA KIAA1199#1 and shRNA KIAA1199#2) CaSki cells were untreated or stimulated with TNFα (200U/ml) for 24 hours and cells in early or late apoptosis were quantified by FACS analyses. A representative FACS experiment carried out with control versus KIAA1199-depleted CaSki cells (shRNA KIAA1199#2) is illustrated. FACS data from three independent experiments are showed in the histogram. (means $\pm$ standard deviations, Student t-test p-values: ***< 0.001).

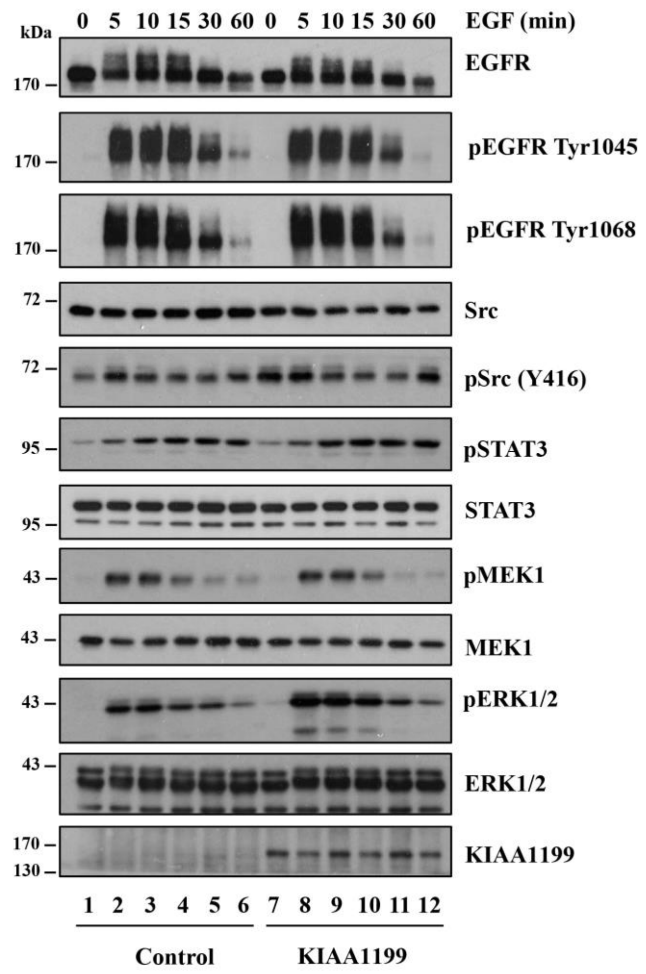

Supplementary Figure 9: KIAA1199 overexpression in SiHa cells potentiates EGF-dependent ERK1/2 but not EGFR phosphorylation. Control or KIAA1199-overexpressing SiHa cells were untreated or stimulated with EGF (100 ng/ml) for the indicated periods of time. The resulting cell extracts were subjected to WB analyses using the indicated antibodies.

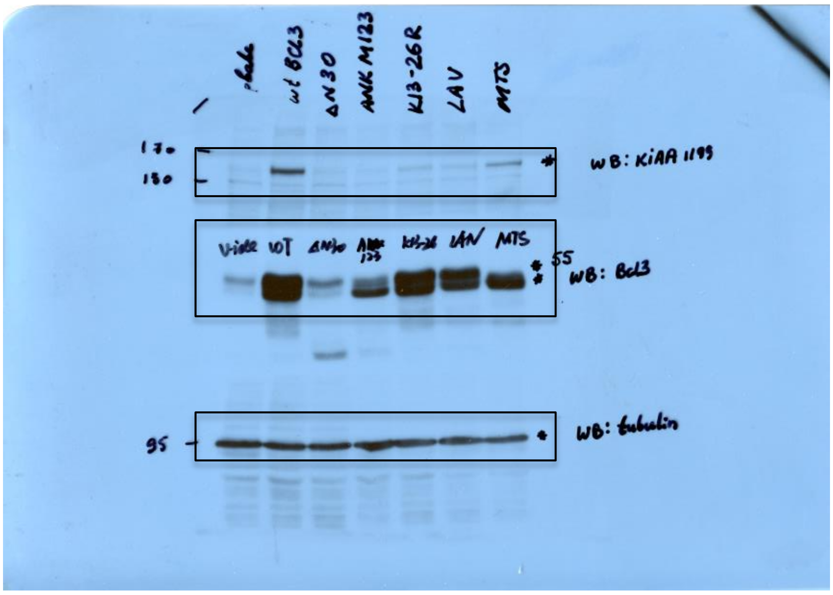

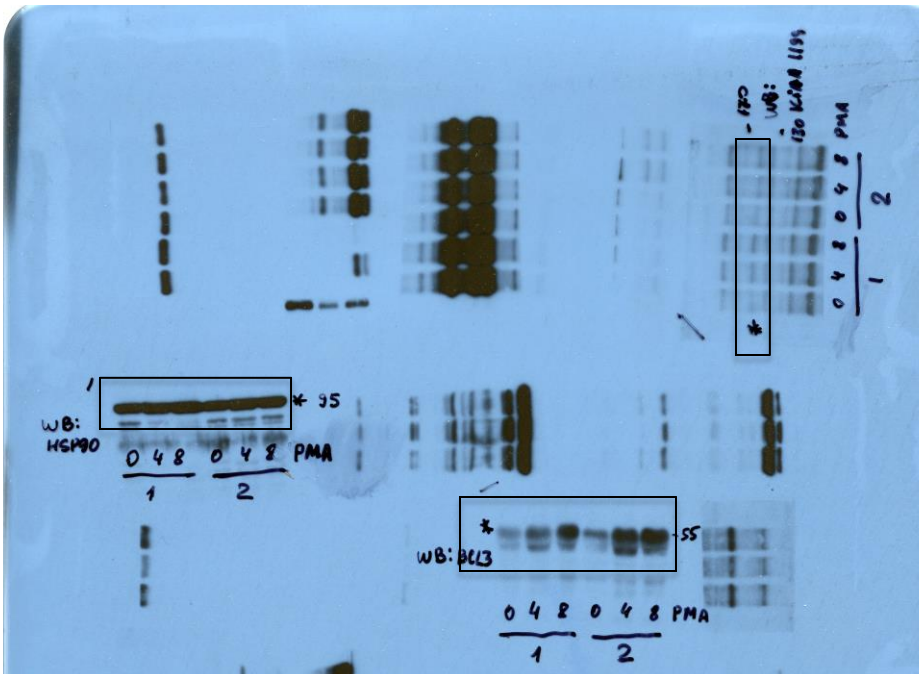

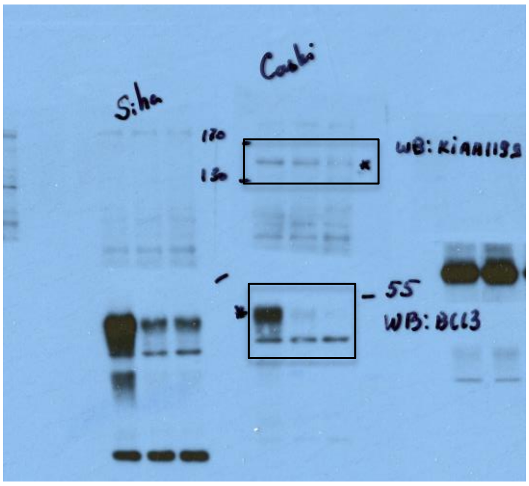

Supplementary Figure 10: Uncropped scans for the main western blots illustrated in Figure 1 of the manuscript. The panels illustrated in the paper are encircled. Top panel: scans illustrated in Figure 1C, bottom panels on the left and on the right: scans illustrated in Figures 1D and 1G, respectively.

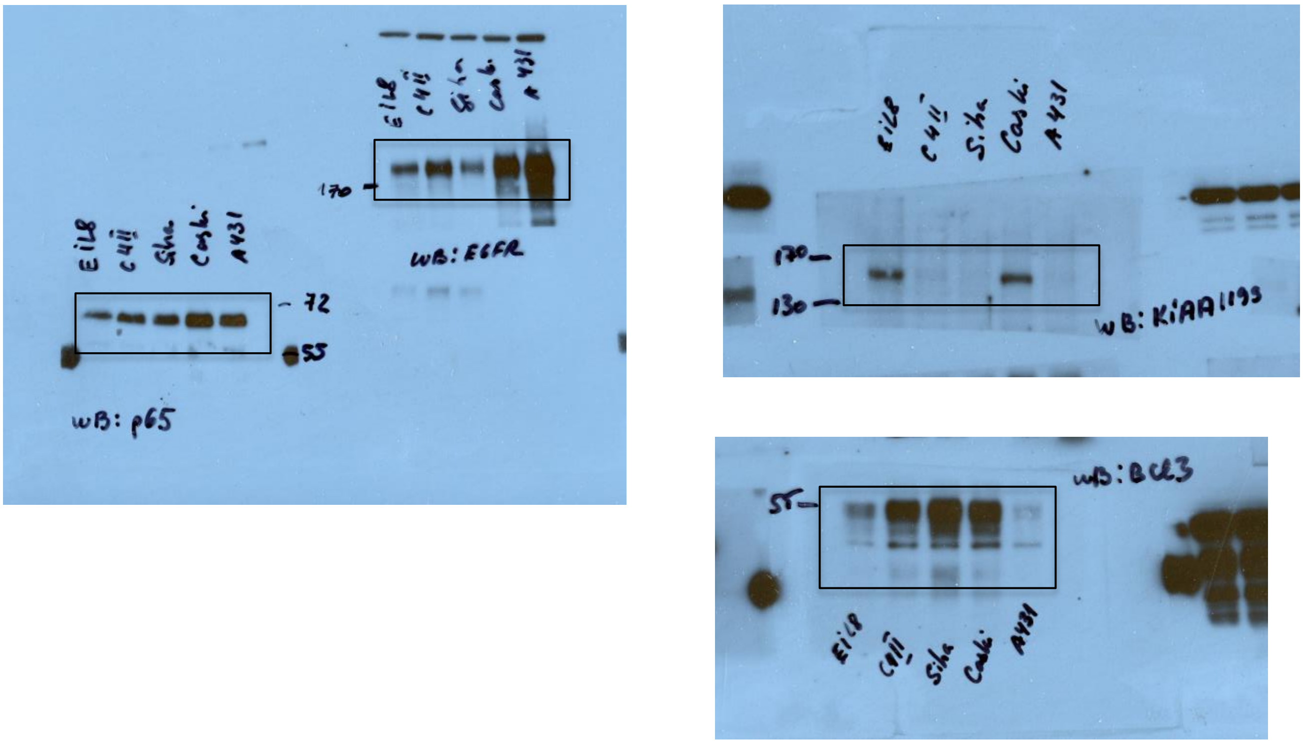

Supplementary Figure 11: Uncropped scans for the main western blots illustrated in Figure 2 of the manuscript. The panels illustrated in the paper are encircled. Panels are shown in Figure 2A of the paper.

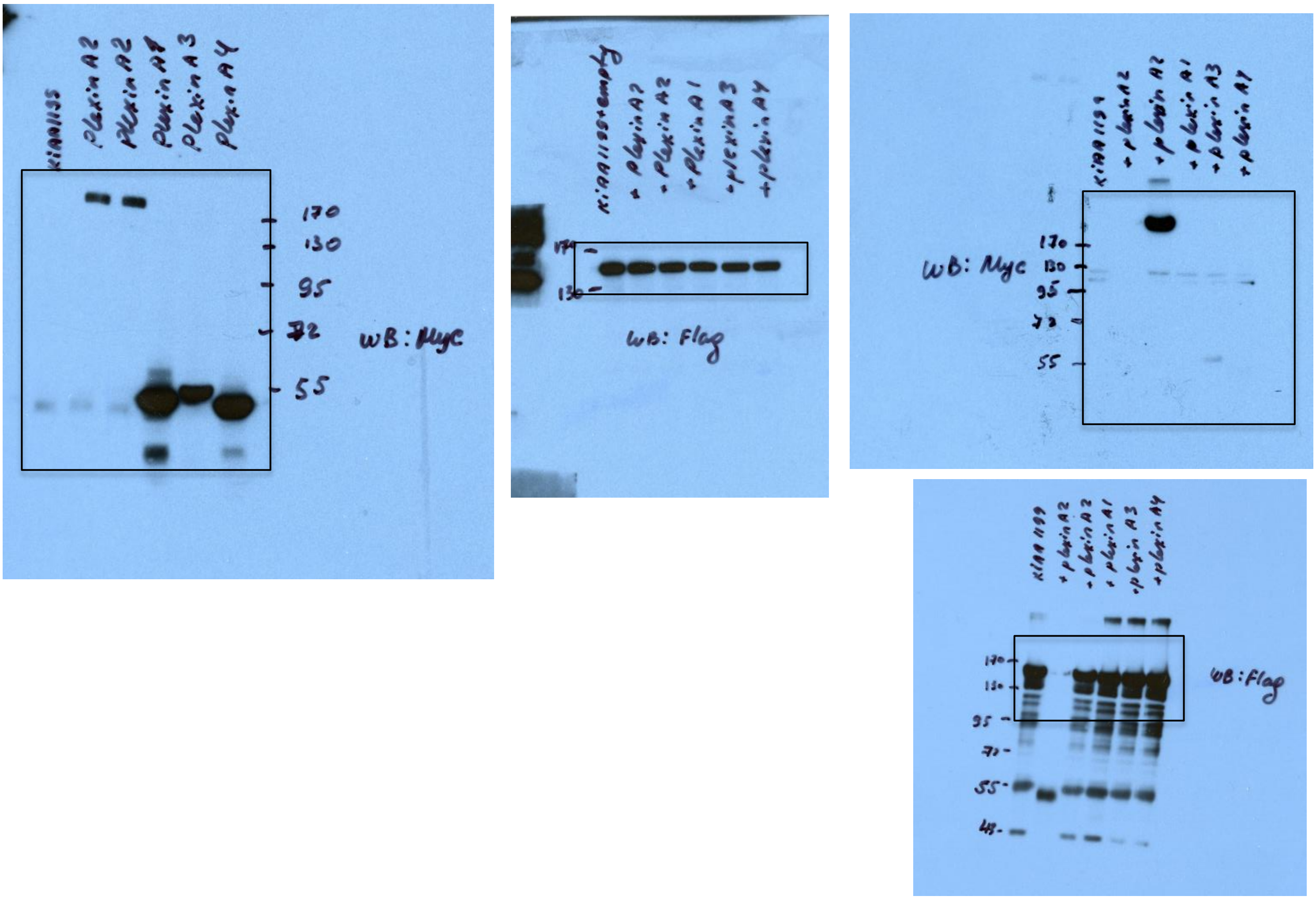

Supplementary Figure 12: Uncropped scans for the main western blots illustrated in Figure 3 of the manuscript. The panels illustrated in the paper are encircled. All panels are illustrated in Figure 3A of the paper.

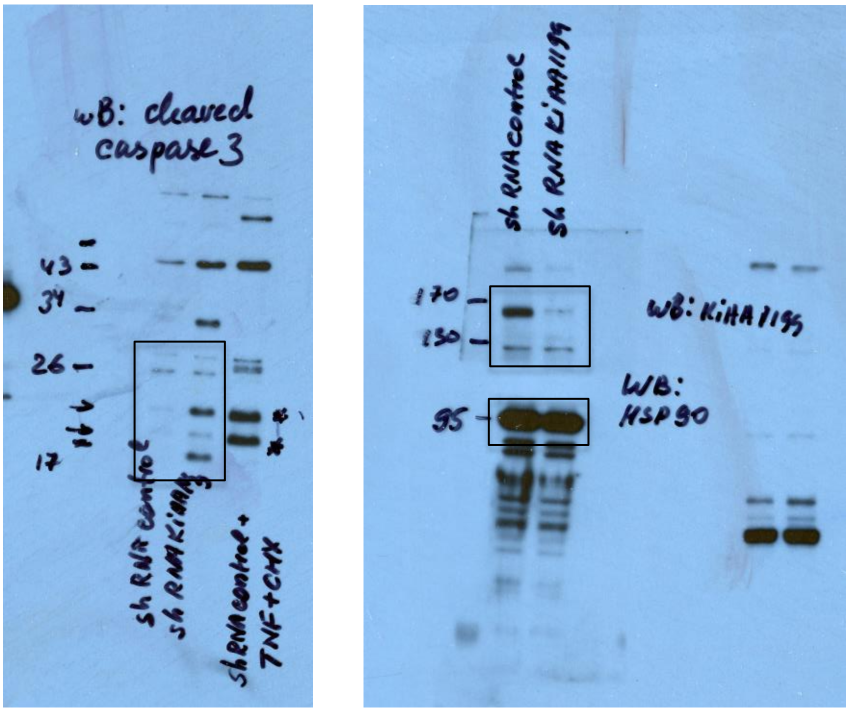

Supplementary Figure 13: Uncropped scans for the main western blots illustrated in Figure 4 of the manuscript. The panels illustrated in the paper are encircled. All panels are illustrated in Figure 4D of the paper.

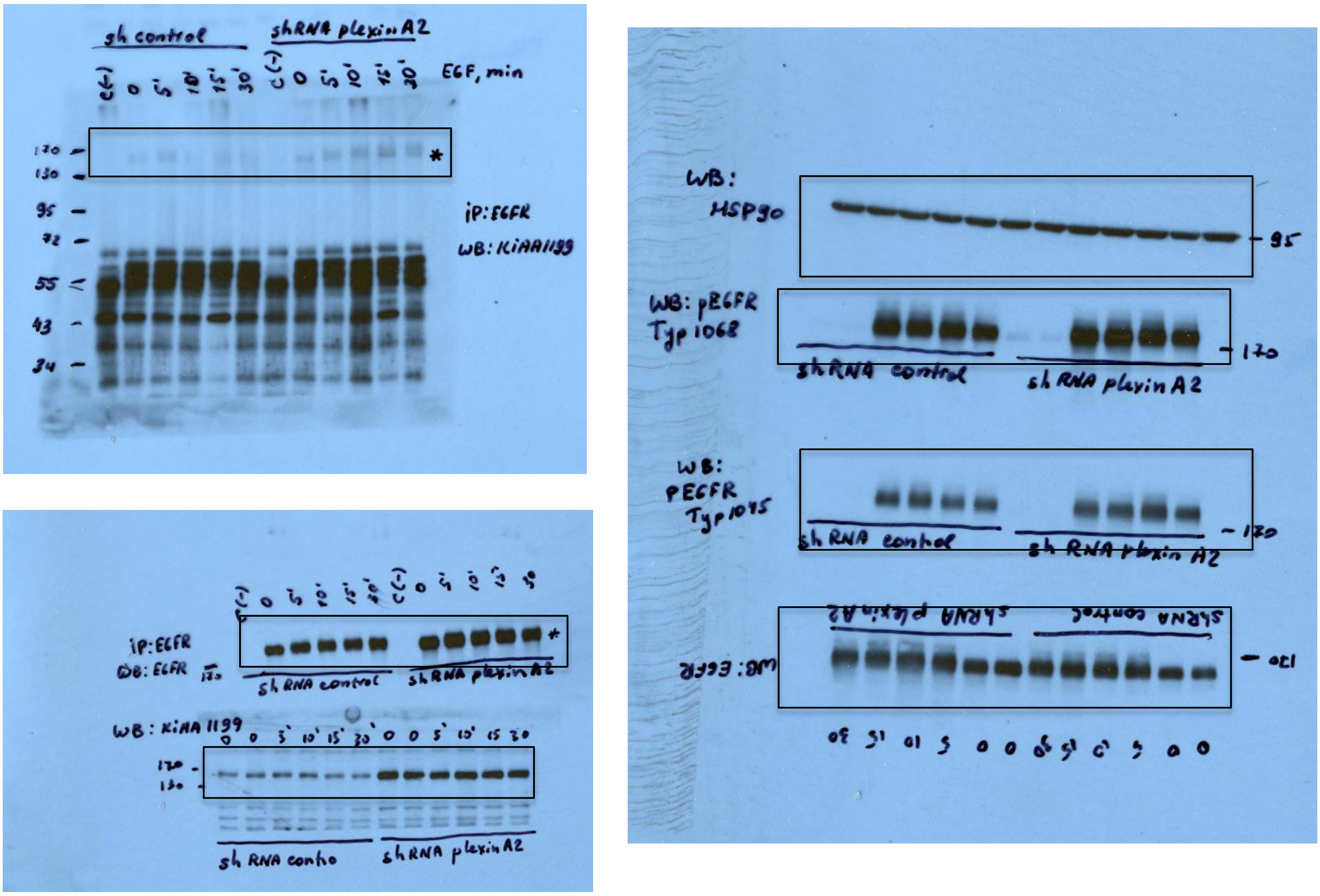

Supplementary Figure 14: Uncropped scans for the main western blots illustrated in Figure 5 of the manuscript. The panels illustrated in the paper are encircled. All panels are illustrated in Figure 5D of the paper.

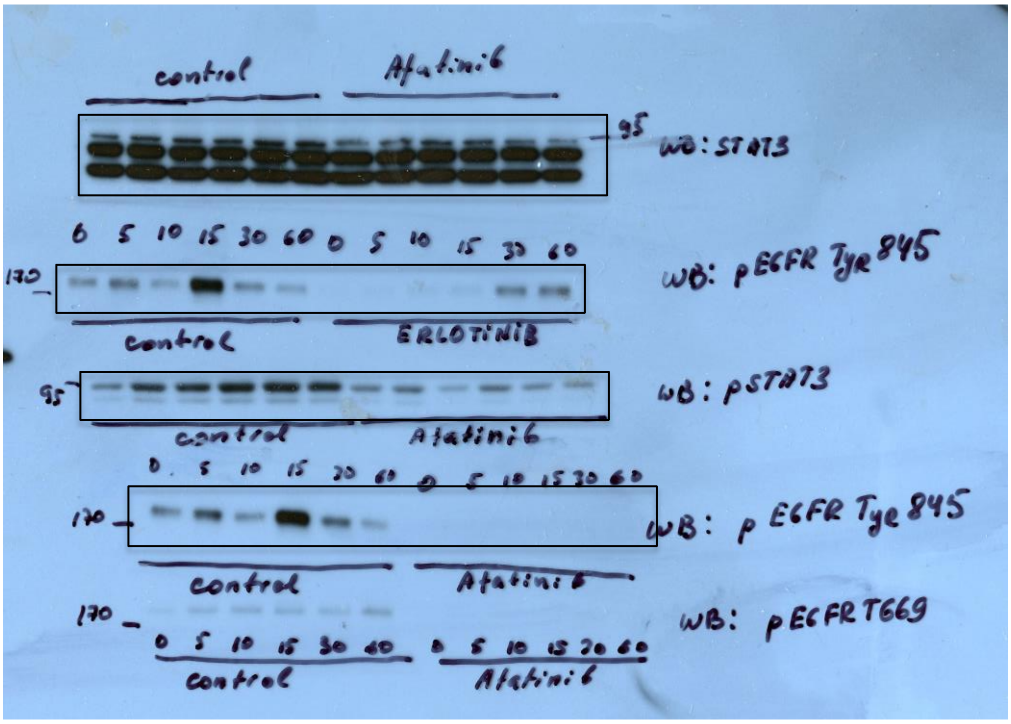

Supplementary Figure 15: Uncropped scans for the main western blots illustrated in Figure 6 of the manuscript. The panels illustrated in the paper are encircled. All panels are illustrated in Figure 6A of the paper.

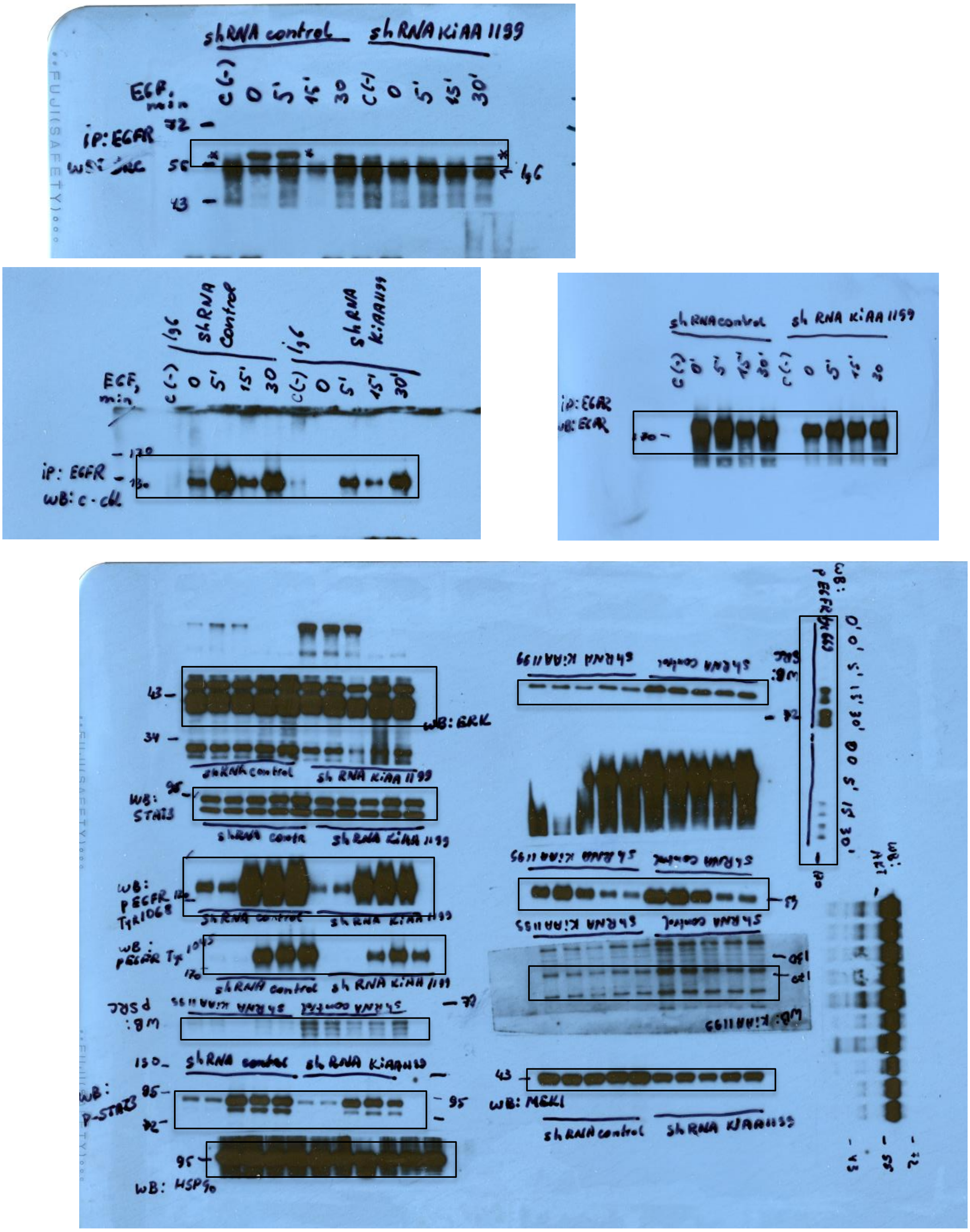

Supplementary Figure 16: Uncropped scans for the main western blots illustrated in Figure 7 of the manuscript. The panels illustrated in the paper are encircled. All panels are illustrated in Figure 7A of the paper.

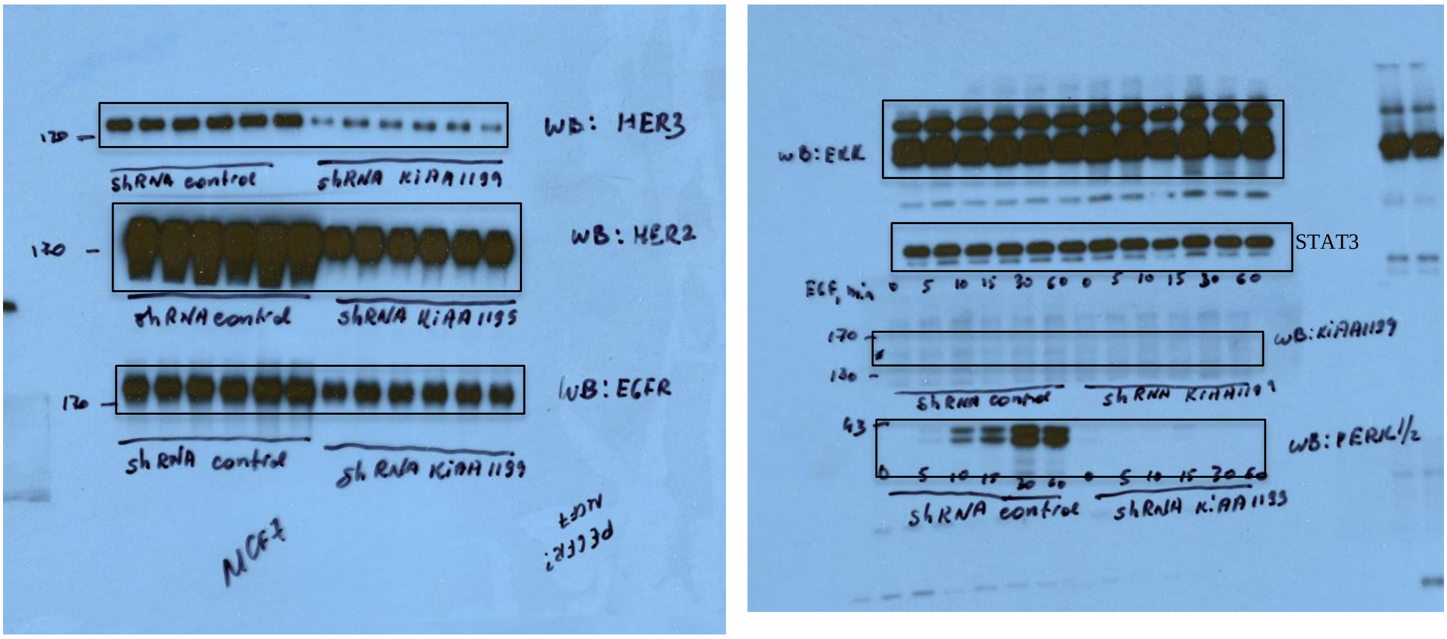

Supplementary Figure 17: Uncropped scans for the main western blots illustrated in Figure 8 of the manuscript. The panels illustrated in the paper are encircled. All panels are illustrated in Figure 8A of the paper.

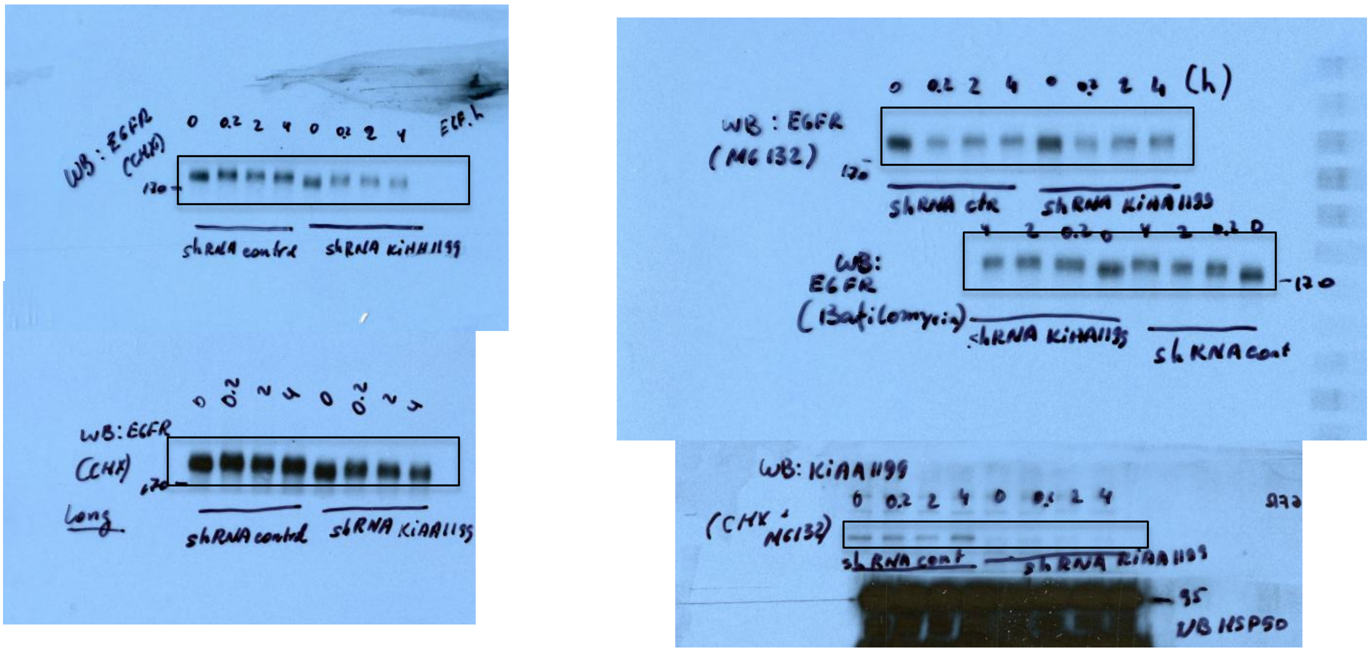

Supplementary Figure 18: Uncropped scans for the main western blots illustrated in Figure 9 of the manuscript. The panels illustrated in the paper are encircled. All panels are illustrated in Figure 9A of the paper.

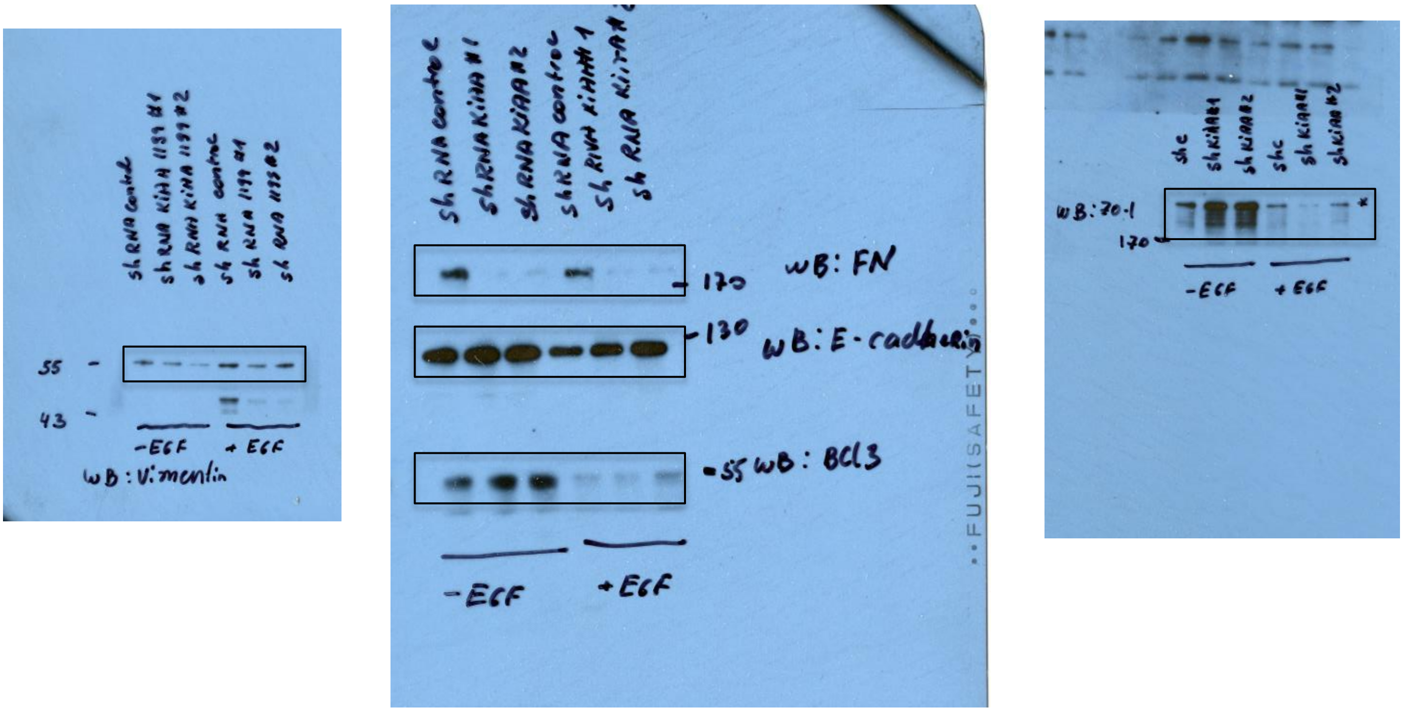

Supplementary Figure 19: Uncropped scans for the main western blots illustrated in Figure 10 of the manuscript. The panels illustrated in the paper are encircled. All panels are illustrated in Figure 10A of the paper.

	Forward (5'-3')	Reverse (5'-3')
κb site1	GCCTCTTCTGTGTCCAACAA	TGGCATCAGAAAGGGAAAT
κb site2	ATGCAAAGAATGGAGGAAGG	CTCCAGAGAGAAGGGAAACG
κb site3	CCCTGCCTTTACCACTTCAT	ACATGCAGGATTTTCCATCA
κb site4	CTCCTACCCACAGGGAATGT	GGTTCTGTGATGCTTCCTCA
intron2	CCGTGTCTGTGTGTGTGCAT	CTGAAAGAGAAGCACACAGA
A (-500bp)	GGCTACTCTTTTGCTGCTGA	GTCCCAGGCTAAGTCTCAGG
B (+1bp)	AGCTAGCGCTCAAGCAGAG	GCCAGGCTCTGTCCGATA
C (+500bp)	GAGGAGGGAGTACCACAAGG	AGGGAAACTGGGAGGAAGAG
D (+1000bp)	ATACCAGGGACGGTGAGC	CCTGGATCACTTTCCAGACC

Table 1: Primer pairs used for Chromatin IP experiments.

	Forward (5'-3')	Reverse (5'-3')
18s	AACTTTCGATGGTAGTCGCCG	CCTTGATGTGTAGCCGTTT
KIAA1199	ACGACGAGCCGATTGTTT	CCTTCATCAGCCCTTCCA
BCL-3	AGACACGCCTCTCCATATTGCT	CCCTGCTGGAAGAGGTTGAC
Plexin A1	ACAGACATCCACGAGCTGACCAAT	AACAGTGTCAGCGACTTCTCCACA
Plexin A2	GTTTGACATCCACAAGGGCAGCAT	AGCTTGGCGATGTCTGCGTAGTAT
Plexin A3	TCTCAACTGTGACAGCATCACCCA	TCACACTCGATCTTGGTGGTGACA
Plexin A4	TTCATGGACTCTTGCTCCACGTCA	TGATGGCTGGCATCTTCCCTATGT
Plexin B1	ACACCATCGCTTTCCTGGGTGATA	AACCTTCAGAAGTGTGCTCTGGGT
Plexin B2	AGAGGATGCTGTCCAACTGGATGT	TGTCGTTGAGAGTGTACTTGGCCT
Plexin B3	TGGTGGAGAAACTGCTCACCAACT	TATCATTCAGGGTCCGTTTGGCCT
Plexin C1	AGCAGGGAGCTCTGCCAGAATAA	CAGTTTCTACGCATGCTGGGCATT
Plexin D1	AAGGATTCGCCAACCAACAAGCTC	CCATGGCCACATTGGTGTTGAACT
E6	CTGCAATGTTTCAGGACCCA	TCATGTATAGTTGTTTGCAGCTCTGT
IL-8	GAAGGAACCATCTCACTGTGTGTA	ATCAGGAAGGCTGCCAAGAG
NRG1	CTGTGTGAATGGAGGGGAGT	CGTAGTTTTGGCAGCGATCA
NRG3	TACCACCACCACACCAGAAA	GGGTTTGAAGTGCTCGGATC
HBEGF	CAAGAGGCAGATCTGGACCT	GCTCCTCCTTGTTTGGTGTG
Tgf alpha	AATGACTGC CCAGAT TCCCA	GCACCAACGTACCCAGAATG
AREG	AGCACCTGGAAGCAGTAACA	GACTTTTCCCCACACCGTTC

Table 2: Primer pairs used for Real-time PCR experiments.
